# Development and comparative investigation of structural and functional properties of Ni-based metal–organic frameworks designed for photocatalytic hydrogen production

**DOI:** 10.1039/d6ra08963a

**Published:** 2026-09-28

**Authors:** Antti Marttinen, Manu Lahtinen

**Affiliations:** a Department of Chemistry, University of Jyväskylä Jyväskylä Finland manu.k.lahtinen@jyu.fi

## Abstract

Designing efficient, economical and stable photocatalysts utilizing the vast potential of sunlight remains a challenge, but at the same time it presents a valuable pursuit in developing alternative renewable energy solutions. Guided by this objective we synthesized and characterized two new metal–organic frameworks (MOFs), namely 1 (Ni-DIA-TBAPY, DIA = 9,10-di(1*H*-imidazol-1-yl)anthracene, TBAPY = 1,3,6,8-tetrakis(*p*-benzoic acid)pyrene) and 2 (Ni-DIP-TBAPY, DIP = 1,6-di(1*H*-imidazol-1-yl)pyrene) designed to act as photocatalysts. Structural analyses using single-crystal X-ray diffraction (SCXRD) revealed their practically identical secondary building units. However, their 3D-frameworks differ significantly, with one exhibiting a flexible structure and the other a rigid one. Both structures demonstrate chemical and thermal stability by maintaining framework integrity even in boiling water, but the flexible structure of 1 exhibits unusual solvent-induced reversible crystalline-to-amorphous transition. Based on their similar optical properties which proved their ability to utilize visible light, their photocatalytic performance in the hydrogen evolution reaction (HER) was investigated. Under simulated AM 1.5G solar irradiation both MOFs proved to be active photocatalysts without any cocatalysts. However, their performance differed significantly in their long-term stability, with 1 partially degrading over consecutive reaction cycles, while 2 remained completely stable without significant loss in photocatalytic activity, proving its better applicability.

## Abstract 2

Synthesis and characterization of two new nickel-based metal–organic framework (MOF) structures along with their comparative investigation of structural stability and activity in photocatalytic hydrogen production.

## Graphical abstract

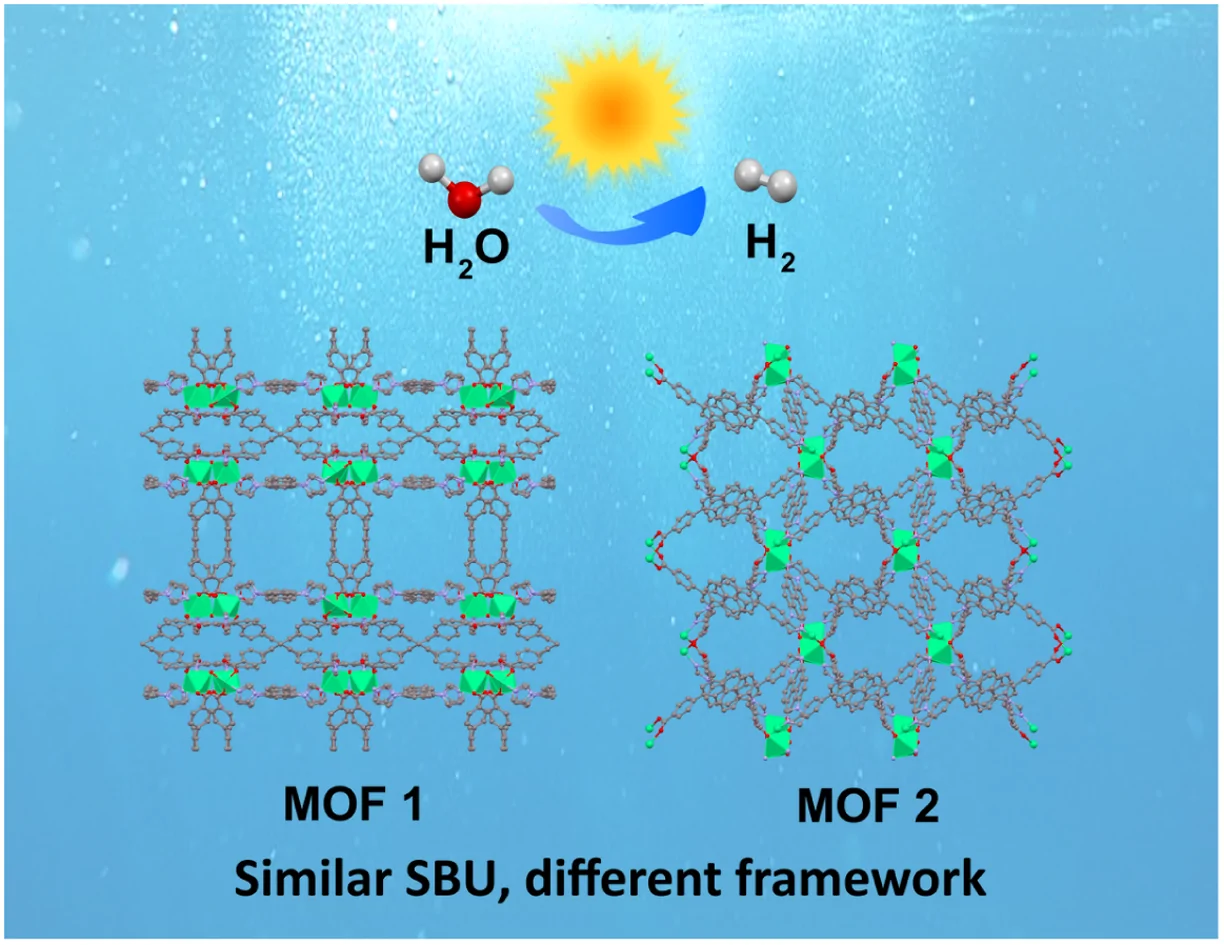

## Introduction

The combined use of natural sunlight and synthetic photocatalysts represents a promising strategy for the development of new alternative technologies such as for water purification^1^ or solar fuel production^2,3^ in the future. Photocatalytic hydrogen production or water splitting are especially attractive approaches of generating renewable energy in the form of hydrogen, utilizing the vast potential of sunlight and high abundance of water. These processes, inspired by natural photosynthesis, could assist in reducing the dependence on fossil fuels and furthermore could achieve that without costly electricity generation. However, significant challenges remain for large-scale implementation, particularly with respect to enhancing photocatalytic efficiencies and material stability.^2–4^ Metal–organic frameworks (MOFs) have been extensively studied for a wide range of applications, including heterogeneous photocatalysis. The modifiability of MOF structures is an advantage in creating large specific surface areas, high porosity and catalytically active open metal sites or functional groups which are beneficial techniques in creating efficient (photo)catalysts. Lot of progress has been made in developing active MOFs for photocatalytic hydrogen production, but challenges remain, with one prevalent among MOFs being their stability in water.^2,3,5^ Since aqueous photocatalytic applications require MOF products to be immersed in water or aqueous solutions they need exceptional hydrolytic stability. Although research efforts in this regard have gained more attention, the number of water-stable MOFs is still limited, and thus there is a great demand for their development to enable more practical applications of MOFs.^3,5,6^

In this study two new mixed-ligand MOFs, namely 1 (Ni-DIA-TBAPY) and 2 (Ni-DIP-TBAPY), designed for photocatalytic hydrogen production applications have been synthesized and characterized. These structures employ 1,3,6,8-tetrakis(*p*-benzoic acid)pyrene (TBAPY) as the main ligand. The ligand was particularly chosen for its visible light absorption, charge transfer properties and its proven applicability in numerous photocatalytically active MOF designs.^7–10^ This ligand was then paired with similar planar core containing N-donor ligands namely, 9,10-di(1*H*-imidazol-1-yl)anthracene (DIA) or 1,6-di(1*H*-imidazol-1-yl)pyrene (DIP) to generate mixed-ligand MOFs. The strategy of using these highly aromatic conjugated ligands was to introduce π–π stacking interactions, thereby enhancing light absorption and electrical conductivity in the resulting MOFs while also improving water stability through local hydrophobicity and stronger N-donor ligand–metal bond strength.^11,12^ With the introduction of mixed ligands, the mechanical and hydrolytic stability of MOFs can be enhanced through increased metal coordination and rigidity or possible framework interpenetration/catenation.^13,14^ Therefore with the combination of increased light absorption, charge-transfer properties and local hydrophobicity from the aromatic ligand cores and the additional predicted benefits of increased rigidity and water stability from mixed-ligand strategy, we aimed to generate both water-stable and photocatalytically active MOFs. Nickel was chosen as the metal center for its high earth abundance, excellent catalytic properties, versatile coordination properties, and its use in many successful photocatalysts.^5,7,15^ Furthermore, because of the rarity and high cost of the prevailing noble metal catalysts (*e.g.* Pt, Pd, Ir), the development of noble-metal-free photocatalysts with comparable efficiencies is highly advantageous and in strong demand for the potential future scaleup of photocatalytic processes.^11,16,17^

With the synthesis and characterization of MOFs 1 and 2, it was observed that the 3D mixed-ligand MOFs contain almost identical secondary building units (SBU) but significantly different framework structures with distinct pore sizes and shapes. The smaller secondary ligand and increased pore size in MOF 1 also allowed formation of dangling linker defect inside the pore, unlike in MOF 2. With differing framework structures, chemical and thermal stability testing was conducted for both MOFs, where reversible crystalline-to-amorphous transition was observed for MOF 1, while MOF 2 remained completely unchanged under various conditions. Further studies of their optical properties revealed their similar light absorption capabilities and successful application in photocatalytic hydrogen evolution (HER) reactions, although differing in their long-term stability and activity in the tested reaction conditions.

## Experimental

### Ligand syntheses

DIA ligand was synthesized according to our previously reported synthesis,^18^ with the exception of that the reaction was conducted at a lower temperature of 110 °C. 9,10-Dibromoanthracene (2.57 g, 7.66 mmol), imidazole (2.08 g, 30.6 mmol) and powdered sodium hydroxide (1.22 g, 30.6 mmol) were transferred with 100 mL of dimethylformamide (DMF) to a 250 mL round flask under magnetic stirring. The mixture was heated under reflux at 110 °C for 24 hours. After that, the mixture was cooled down to room temperature and poured into 250 mL of water. The yellow precipitate was filtered, washed with water (2 × 50 mL), toluene (2 × 50 mL) and hexane (1 × 50 mL) to obtain a pale yellow powdery product which was then dried in a desiccator. Yield 1.16 g, 49%. ^1^H NMR (Fig. S1) *δ* 7.83 (d, 2H), 7.52–7.57 (m, 8H), 7.49 (s, 2H), 7.33 (d, 2H). PXRD analysis is presented in Fig. S4.

DIP ligand was synthesized by a method which was inspired by the solvent free synthesis of 1,4-di(1*H*-imidazol-1-yl)benzene by Dong *et al.*^19^ 1,6-dibromopyrene (1.00 g, 2.78 mmol), imidazole (0.76 g, 11.2 mmol), K_2_CO_3_ (1.55 g, 11.2 mmol) and CuI (0.16 g, 0.84 mmol) were mixed together and loaded into a 45 mL PTFE-lined steel autoclave (without any solvent). The autoclave was closed and heated at 160 °C for 18 hours. After cooling down to roughly 50 °C, the crude product was stirred in 100 mL of H_2_O for 15 minutes. Product was filtered and washed with H_2_O, acetonitrile, toluene and lastly diethyl ether. Then brownish product was recrystallized from 150 mL of hot chloroform and dried in a desiccator to yield colorless/slightly beige powder. Yield 0.72 g, 77%. ^1^H NMR (Fig. S2) *δ* 8.31 (d, 2H), 8.19 (d, 2H), 8.04 (d, 2H), 7.91–7.95 (m, 4H), 7.41 (br, s, 4H). Crystallographic structure examination and PXRD analysis (Fig. S5) are presented in the SI.

### MOF single-crystal crystallizations and syntheses

#### Synthesis of 1

Dia (22.0 mg, 0.07 mmol), TBAPY (24.3 mg, 0.04 mmol), NiOAc·4H_2_O (37.3 mg, 0.15 mmol) were loaded into a 22 mL PTFE-lined steel autoclave with 5 mL of DMF, 2.5 mL of 1,4-dioxane and 2.5 mL of H_2_O. Mixture was briefly stirred before sealing the autoclave and heating it at 120 °C for 48 hours and then cooled to room temperature (RT) at a rate of ∼4 °C h^−1^. Light green crystals suitable for SCXRD were selected from the resulting mixture. Isostructural dry structure of 1B was obtained from the same crystals dried in RT. Synthesis of 1 was further modified by doubling the amount of starting materials and using a component ratio of 2 : 1 : 6 (DIA : TBAPY : NiOAc) to yield pure light green powder phase of MOF 1 after suction filtration and drying the product in a desiccator.

#### Synthesis of 2

DIP (49.7 mg, 0.15 mmol), TBAPY (50.4 mg, 0.07 mmol), NiOAc·4H_2_O (72.9 mg, 0.29 mmol) were loaded into a 22 mL PTFE-lined steel autoclave with 5 mL of DMF, 2.5 mL of 1,4-dioxane and 2.5 mL of H_2_O. Mixture was briefly stirred before sealing the autoclave and heating it at 120 °C for 48 hours and then cooled to room temperature (RT) at a rate of ∼4 °C h^−1^. Light green/colourless crystals suitable for SCXRD were selected from the resulting mixture of crystals. Synthesis of 2 was further modified using component ratio of 2 : 1 : 6 (DIA : TBAPY : NiOAc), doubling the solvent amounts (and autoclave size) and increasing reaction time to 72 hours. Product was washed with hot H_2_O and dried in a desiccator to yield pure light green powder.

### Single-crystal X-ray diffraction (SCXRD)

Single-crystal data was measured with Rigaku XtaLAB Synergy-R single source high flux rotating anode X-ray diffractometer equipped with HyPix-Arc 100° detector and multilayer optics producing monochromatized Cu Kα (1.54187 Å) radiation. Data collection, reduction, and analytical face-index-based absorption correction of the data were carried out using the CrysAlisPRO program (v.42).^20^ All structures were solved by intrinsic phasing (ShelXT^21^) and refined on *F*^2^ by full-matrix least-squares techniques with the ShelXL^22^ subprogram in the Olex2 (ref. 23) (v.1.5) structure solving program that utilizes the SHELXL-2013 module. All C–H hydrogen atoms were calculated to their optimal positions and treated as riding atoms using isotropic displacement parameters of 1.2 (sp^2^ group) larger than the host atom. Disordered solvent molecules were first identified based on electron density maps and their geometrical features, but due to severity of the disorder they were excluded in the final refinement by Olex2 solvent mask (1.2 Å solvent probe radius). Identification and assumptions of disordered structural fragments of ligands, molecules or atoms were based on the reagents used in the synthesis, the residual electron densities, and their geometrical features. Occupancies of the disordered atoms were initially refined freely and then fixed accordingly to equivalent 0.5 occupancies or in select cases to more precisely refined values *e.g.* 0.25, 0.3, 0.45 or 0.7. CrystalNets^24^ and TopCryst^25^ interfaces were utilized in topology determination. Crystallographic data with selected bond distances and angles are presented in the SI.

### Powder X-ray diffraction (PXRD)

Powder products were analyzed with Panalytical X'Pert Pro MPD diffractometer with Cu Kα radiation (*λ* = 1.54187 Å; Ni β-filter; 45 kV, 40 mA). Powder samples were prepared on a silicon-based zero background generating plate using petroleum jelly as an adhesive. Position-sensitive X'Celerator detector was used to record the diffraction intensities at room temperature with a 2*θ* range of 2–60°, a step size of 0.017°, and a counting time of 50–280 s per step. Data processing, pattern comparisons and Pawley whole-pattern refinements^26^ were carried out with the X'Pert HighScore Plus^27^ (v. 4.9) software. In the Pawley refinements, the corresponding single-crystal structure parameters were used as the basis for the least-squares refinement of the unit cell parameters of the PXRD patterns. ICDD PDF5+ powder diffraction database^28^ implemented in the HighScore software was used for qualitative search-match phase analysis for examining potential known impurities and side-products.

### Photocatalytic hydrogen production experiments

Photocatalytic hydrogen evolution reactions (HER) were conducted in 23 mL low-potassium borosilicate glass reaction vials containing 10 mL of solution and a 13 mL headspace. First 10 mg of MOF powder was dispersed in 10 mL of H_2_O : MeOH (1 : 1). 26 mg (0.15 mmol) of ascorbic acid was added to the solution (MeOH and ascorbic acid acting as sacrificial donors) and the vial was sealed with a silicone septum. Mixture was degassed by bubbling Argon through the solution for 15 minutes. Suspension was magnetically stirred and irradiated by a LED AM 1.5G solar simulator (G2009A1, Ossila) in the 380–1000 nm wavelength range with irradiance value of 100 mW cm^−2^. Irradiance value was measured using a optical power meter (Thorlabs, PM-100D with a S401C sensor). Temperature of the reactions was 25–30 °C due to slight system heating during irradiation. Gas samples (200 µl) were collected from the vial headspace and analysed using an Agilent 8860 gas chromatograph equipped with a PLOT Molesieve 5 Å column, a thermal conductivity detector (TCD) and argon as carrier gas. Apparent quantum efficiency (AQE) measurements were conducted under identical conditions except for using 450 nm LED light instead of simulated solar spectrum. Full details of the GC analysis and AQE calculations are presented in the SI.

The longer photocatalytic cyclic tests were performed under identical conditions; however, after each cycle the mixture was allowed to settle overnight. The supernatant solution was then withdrawn from above the separated powder, and the remaining solution was evaporated to dryness in an oven at 100 °C. The recovered powder was then dispersed back in a fresh reaction solution (as described above) for the subsequent reaction cycle.

### Other analytical methods

The ^1^H NMR spectra were measured using Bruker Avance III 300 MHz spectrometer with deuterated chloroform (CDCl_3_) as solvent while using residual CHCl_3_ signal (7.26 ppm) as internal standard. UV-vis diffuse reflectance spectra of the MOF powders were recorded using a PerkinElmer Lambda 850 UV-vis spectrometer equipped with an integrating sphere. Powders were placed between two glass slides which was then attached in the integrating sphere center mount. Two empty glass slides along with the center mount were accounted for in the baseline correction. Thermogravimetric analyses (TGA) were conducted using PerkinElmer STA 6000 simultaneous TG/DSC thermal analyzer with samples heated at a rate of 10 °C min^−1^ in the range of 20–600 °C under air flow. N_2_ adsorption isotherms were collected at an analysis temperature of 77 K using Anton Paar Autosorb 6100 FKM XR instrument. Samples were degassed under dynamic vacuum at 100 °C for 12 h prior to analysis. Specific surface area calculations were based on the Brunauer–Emmett–Teller (BET) method in the linear relative pressure range. Scanning electron microscopy (SEM) images were recorded using Zeiss EVO-50 instrument.

## Results and discussion

### Structural description of 1

Compound 1 crystallizes in the orthorhombic space group of *Ibam* (no. 72). The asymmetric unit contains two halfs of TBAPY ligand and two and a half DIA ligands coordinated to two nickel atoms. The two nickel atoms form a secondary building unit (SBU) by their octahedral coordination and bridge connection to each other through a coordinated water molecule (Fig. 1a). The octahedral coordination geometry of nickel atoms is highly symmetrical with just minor deviations in bond lengths and angles (Table S4). Carboxylate groups of the TBAPY ligands coordinate to nickel either monodentatively where the noncoordinating oxygen atom is then hydrogen bonded to water; or bridging bidentatively to two neighbouring Ni-atoms. The DIA ligands have adopted the *cis*-conformation and the imidazole rotation with respect to the anthracene plane varies between 69.4–87.5°. Unusually, one of the DIA molecules occupies some of the structure's void space as a dangling linker defect (Fig. 1b) and it is disordered over an inversion centre along with positional disorder with water and a DMF molecule. Nevertheless, the components create a porous 3D-MOF structure (Fig. 1c) where DMF, water and 1,4-dioxane molecules are highly disordered inside the channels (largest channel size along *a*-axis ∼16.3 × 12.4 Å) of the MOF along with the dangling ligand. The total solvent accessible volume was calculated as 21736 Å^3^ (48% of unit cell) containing 4704 electrons corresponding to 64H_2_O, 80 DMF and 16 1,4-dioxane molecules (4608 e^−^) per unit cell. Topologically the structure 1 can be defined as a [4,7]-connected network, where the tetratopic TBAPY ligand nodes connect four SBUs together, while the 8-connected SBU nodes (Fig. 1a) extend the framework in 7 spatial directions due to the single terminal DIA ligand in the SBU.

**Fig. 1:**
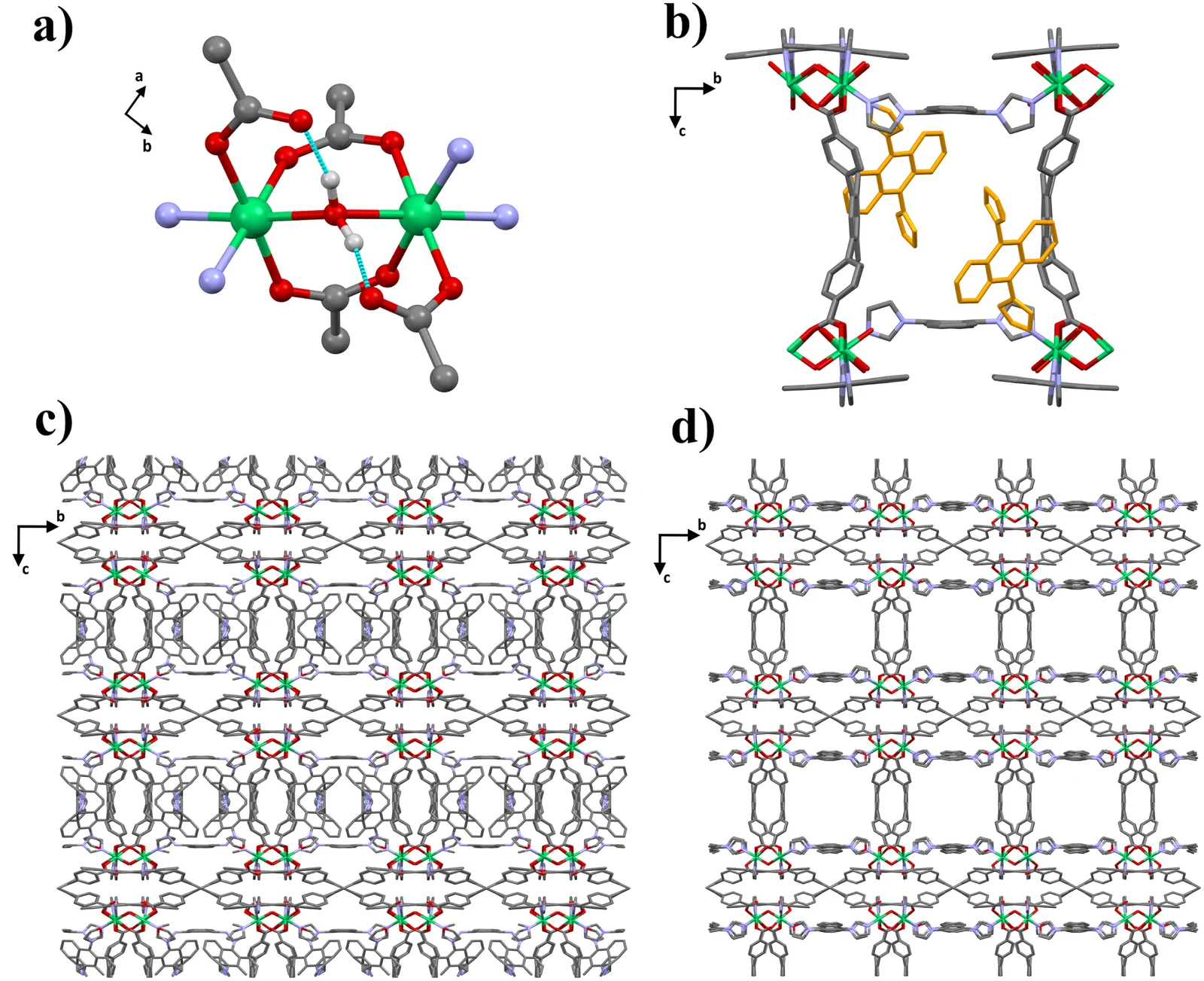
(a) Representation of the SBU of 1, light blue lines represent hydrogen bonding. (b) dangling DIA-ligands represented in orange occupying part of the void space of 1. (c) 3D-framework of 1 and its channels along the *a*-axis (dangling DIA-ligand disordered over an inversion centre). (d) 3D-framework of 1B and its channels along the *a*-axis. Atom colour codes Ni: green, N: blue, O: red, C: grey, H: white.

Further characterization of the dried crystals of 1 revealed another isostructural structure (1B) where most of the solvents from inside the pores have evaporated (compared to solvent electron density of 4704 e^−^ observed in 1, only 904 e^−^ are found in 1B with solvent accessible volume of 20850 Å^3^ accounting for 45% of unit cell volume). This seems to cause slight decrease in the pore volume (−3%) and orientation twisting of the monocoordinated dangling DIA ligand to a more ordered position aligned with neighbouring dicoordinated DIA ligands (Fig. 1d). Therefore, it seems that as a result of its exceptional rotational freedom, the monocoordinated DIA ligand acts as a void filler along with the solvents and based on the solvents or lack of them, it moves inside the pores to fill the most favourable empty space. Both structures of 1 and 1B exhibit only weak π–π stacking interactions^29–31^ by the neighbouring TBAPY ligand benzene rings with centroid–centroid distances of 3.99–4.06 Å (Fig. S8).

### Structural description of 2

Single-crystal analysis revealed that compound 2 crystallizes in the monoclinic space group of *C*2/*c* (no. 15). Asymmetric unit contains a nickel atom where two crystallographically independent halfs of DIP, one half of TBAPY ligand and one water molecule are coordinated. The coordination geometry around the two neighbouring nickel atoms bridged by a water molecule is identical to 1 (Fig. 2a) and the octahedral geometry is highly symmetrical (Table S6). The DIP ligands are in *trans*-conformation, unlike the DIA ligands in structure 1, and the imidazole rings are rotated 69.8–81.9° relative to the pyrene plane. One of the DIP ligands is disordered in two different orientations, but the structure doesn't contain any unusual dangling ligands as in 1. Since 2 contains identical topological connectivity as 1 (same SBU and tetratopic TBAPY ligand node), with the only exception being the lack of any terminal ligands, it can be topologically described as a [4,8]-connected network. The ligands together with the SBUs create a similar porous structure as 1 except with smaller and different shaped channels (Fig. 2b, largest channel size along *a*-axis ∼5.4 × 16.0 Å) containing the disordered solvent molecules. Total solvent accessible volume was calculated as 2300 Å^3^ (27% of unit cell) containing 488 electrons corresponding to 8 H_2_O, 8 DMF and 2 1,4-dioxane (496 e^−^) molecules per unit cell.

**Fig. 2:**
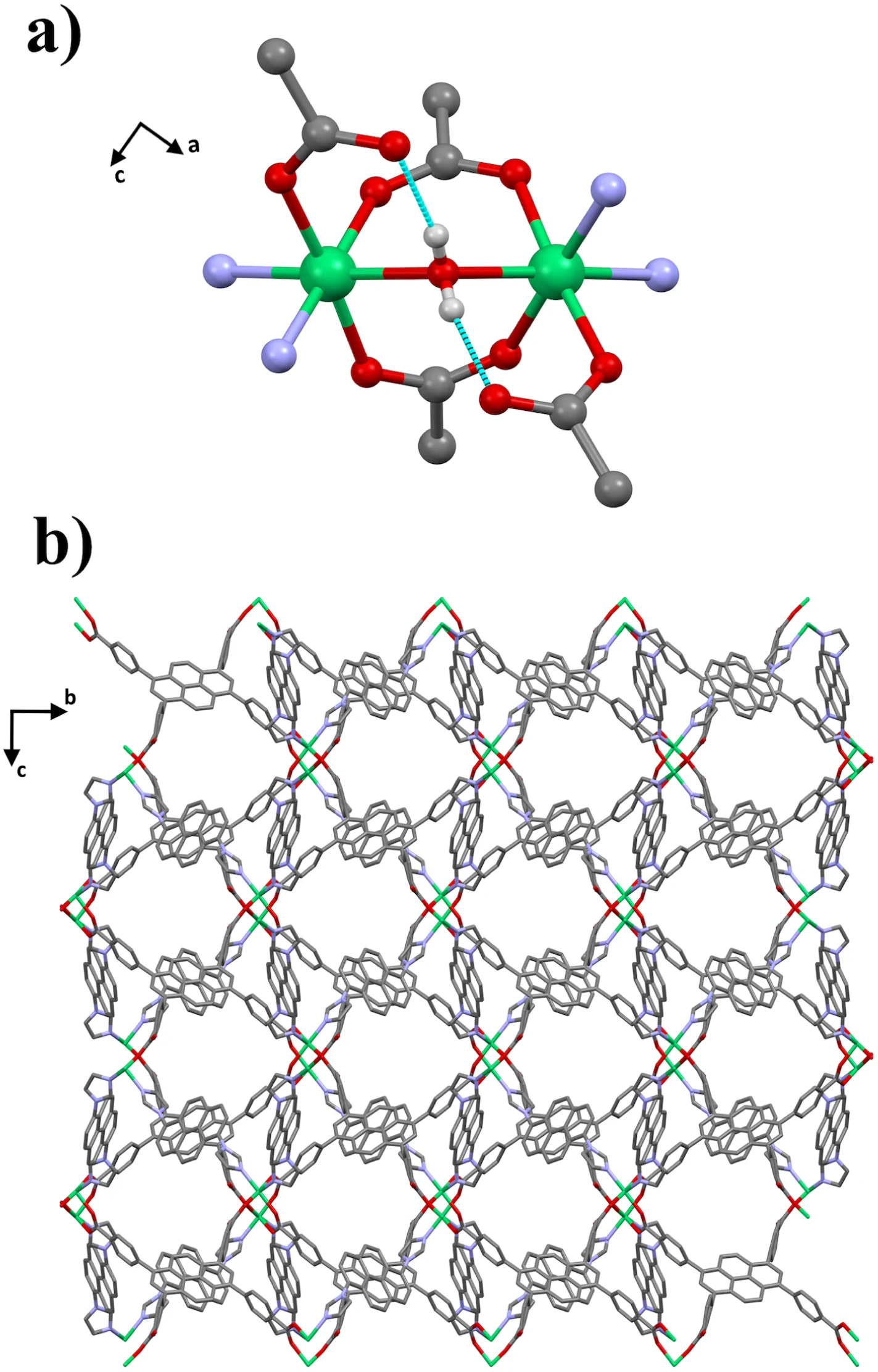
(a) Representation of the SBU of 2, light blue lines represent hydrogen bonding. (b) 3D-framework of 2 and its channels along the *a*-axis. Atom colour codes Ni: green, N: blue, O: red, C: grey, H: white.

The structure also contains another type of channels through which the DIP ligands form a self-catenated 3D-framework of 2 (Fig. S9a). The DIP ligand going through these channels is surrounded by two TBAPY ligands. Together, the pyrene ring of DIP with the benzene and pyrene rings of TBAPY display both π–π stacking interactions and C–H–π interactions with closest centroid–centroid distances of 3.83 Å and H–π distances of 3.24 Å (Fig. S9b). Thus, due to the better size matching of DIP ligand with TBAPY ligand the structure of 2 is seemingly defect-free, more dense and less porous but exhibiting stronger non-covalent interactions, which together could make the structure more stable compared to 1.

### Bulk product syntheses, characterization and stability tests

Based on the single-crystal crystallizations, solvothermal MOF syntheses were slightly modified and optimized to yield pure MOF powder phases for further analysis. Powder X-ray diffraction (PXRD) analysis and Pawley refinements were used to confirm the phase purity of the products and their correspondence to the simulated patterns from SCXRD data (Fig. S10–S13). In the case of 1, it was noticed that the first peak at 3.5° corresponding to indices (0 0 2) disappears when the powder dries (Fig. S10). This kind of behaviour is common with flexible MOF structures.^32–34^ Thus, it can be deduced that the movement of the dangling DIA linker and evaporation of solvents can cause some loss of long-range order in the structure, but the rest of the PXRD pattern matches well with the simulated pattern of 1/1B indicating phase purity (slightly better match with 1B due to its SCXRD measurement done closer to room temperature at 0 °C).

Since water stability is a key prerequisite for successful photocatalyst operating in aqueous media, the stability of synthesized MOFs was tested by immersing and boiling the MOF powders in deionized water or soaking them in aqueous solutions of pH 3 and pH 10. In case of 1 even just immersing the powder in water causes slight color change (from light green to light yellow/yellow green) in the crystals and powder to form 1C. This could be due to complete replacement of the dangling DIA ligand defect with water on the secondary building unit, and so a generation of a missing linker defect, which in this case causes significant structure rearrangement or loss of long-range order in the structure. This is further evidenced by the loss of peaks in the powder pattern of 1 after boiling it in water (Fig. 3a, 1 boiled = 1C) and loss in crystal diffraction when attempting to measure single-crystal data from 1C.

**Fig. 3:**
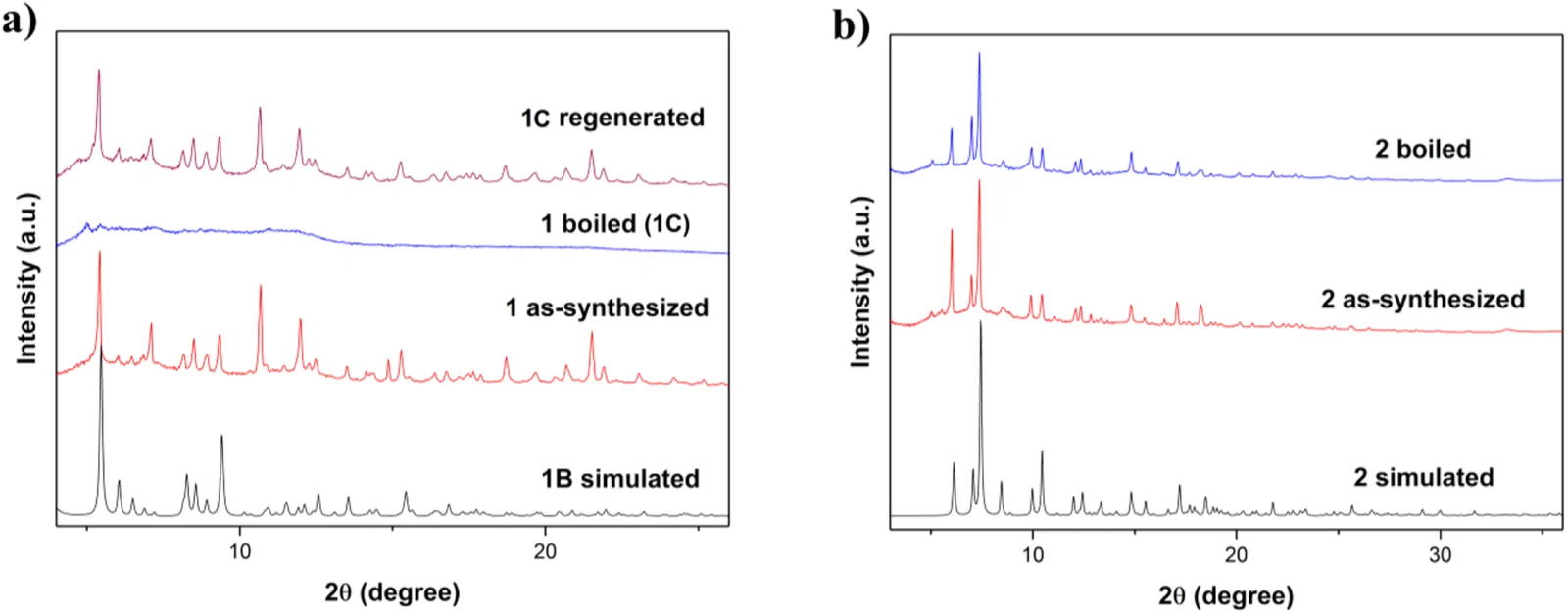
(a) PXRD patterns of 1 before and after boiling water treatment (1 h) along with regenerated phase compared to the SCXRD simulated pattern of 1B. (b) PXRD patterns of 2 before and after boiling water treatment (1 h) compared to the SCXRD simulated pattern of 2.

This amorphization phenomenon was then further examined by immersing the powders or crystals of 1C back in the synthesis solvent mixture (DMF : 1,4-dioxane : H_2_O, 2 : 1 : 1) to observe potential recovery of the structure. It was observed that just by immersing the powders in these solvents wasn't enough to recover the structure completely, but when the boiling water treated product of 1C is heated briefly in the solvent mixture, the structure of 1 can be recovered with a comparable crystallinity to pristine 1 (Fig. 3a). This demonstrates that the framework of 1 exhibits water stability and that the amorphization process of 1 is not caused by the irreversible dissociation of the framework but due to its structural flexibility and distortion which results in loss of long-range order.^34,35^

Therefore, compound 1 seems to have a flexible structure exhibiting a reversible crystalline-to-amorphous transformation, similarly as has been reported in the literature for some MOFs.^33–36^ Most likely in case of 1 this behaviour is due to the movement of the dangling DIA ligand, its disorder, competition with water and solvents along with the possible detachment from metal and reattachment of the dangling DIA with generation of missing linker defect and dangling linker defect, respectively. This is supported by the fact that, if detached from the metal node, the DIA ligand with maximum length of around 11.5 Å would be highly confined inside the channels of 1 (largest possible channel size approximately 16 × 12 Å). This would decrease the possibility of its leaching and would ensure its availability for reforming the dangling ligand coordination after solvent removal from the metal node by heating, as evidenced by the regeneration of the PXRD pattern. Similar reversible behaviour was observed upon soaking 1 in acidic or alkaline aqueous solutions after which, regeneration allowed the recovery of the pattern, demonstrating additional framework stability outside of neutral pH area (Fig. S14).

On the other hand, MOF 2 seems to be completely rigid and stable in water. It can retain its structure and crystallinity even in boiling water (Fig. 3b), possibly due to its smaller pore volumes, larger size of the DIP ligand and lack of any defects which together create a more rigid and ordered structure. Furthermore, the structure retained its integrity upon treatment in acidic or alkaline solutions (Fig. S15).

To get some further insight into the stability and porosity differences between the structures, TGA and BET surface area analyses were conducted. Based on the thermal analyses (Fig. S19), 1 and 2 possess high thermal stability up to 300 °C, with adsorbed or pore solvent evaporation occurring in the range of 20–150 °C. Both structures display very similar thermal degradation initially starting at above 300 °C with final degradation occurring at temperature of around 365 °C. N_2_ adsorption isotherms of activated samples of 1 and 2 (Fig. S20) resulted in estimated BET specific surface areas of 302.5 m^2^ g^−1^ and 102.8 m^2^ g^−1^, respectively, confirming the higher porosity of 1 compared to 2 observed from the SCXRD data. However, it is worth noting that PXRD analysis of the samples after the sorption measurements revealed that 1 transforms into the amorphous 1C during sample activation likely due to the evacuation of the pore stabilizing solvents and subsequent pore contraction which in turn likely reduced the observed specific surface area value, especially due to the presence of DIA ligand defects. On the other hand, the high specific surface area value of 1C provided further evidence of the preservation of the porous framework despite amorphization, especially since the solvent-induced regeneration again allowed the recovery of 1 (Fig. S16). In contrast, MOF 2 remained completely unchanged (Fig. S17), just as was observed in the water stability tests further corroborating structural flexibility differences between the structures and the thermal stability observations from TGA.

### Optical properties and photocatalytic hydrogen evolution

As light absorption and the optical band gap of the MOF powders can have a significant effect on photocatalytic applicability, they were investigated by UV-vis diffuse reflectance spectroscopy (Fig. 4a). Based on the absorption spectra, Tauc analyses and related studies reported in the literature,^8,9,37^ direct electronic transitions were assumed yielding estimated optical band gaps of 2.70 eV and 2.64 eV, for 1 and 2, respectively (Fig. S21). Both the absorption spectra and band gap estimates are well comparable to those reported for a series of TBAPY single-ligand MOFs reported in the literature^8,9,38^ signifying that even with the inclusion of DIA and DIP ligands in 1 and 2, the TBAPY ligand has a dominating role in the light absorption.

**Fig. 4:**
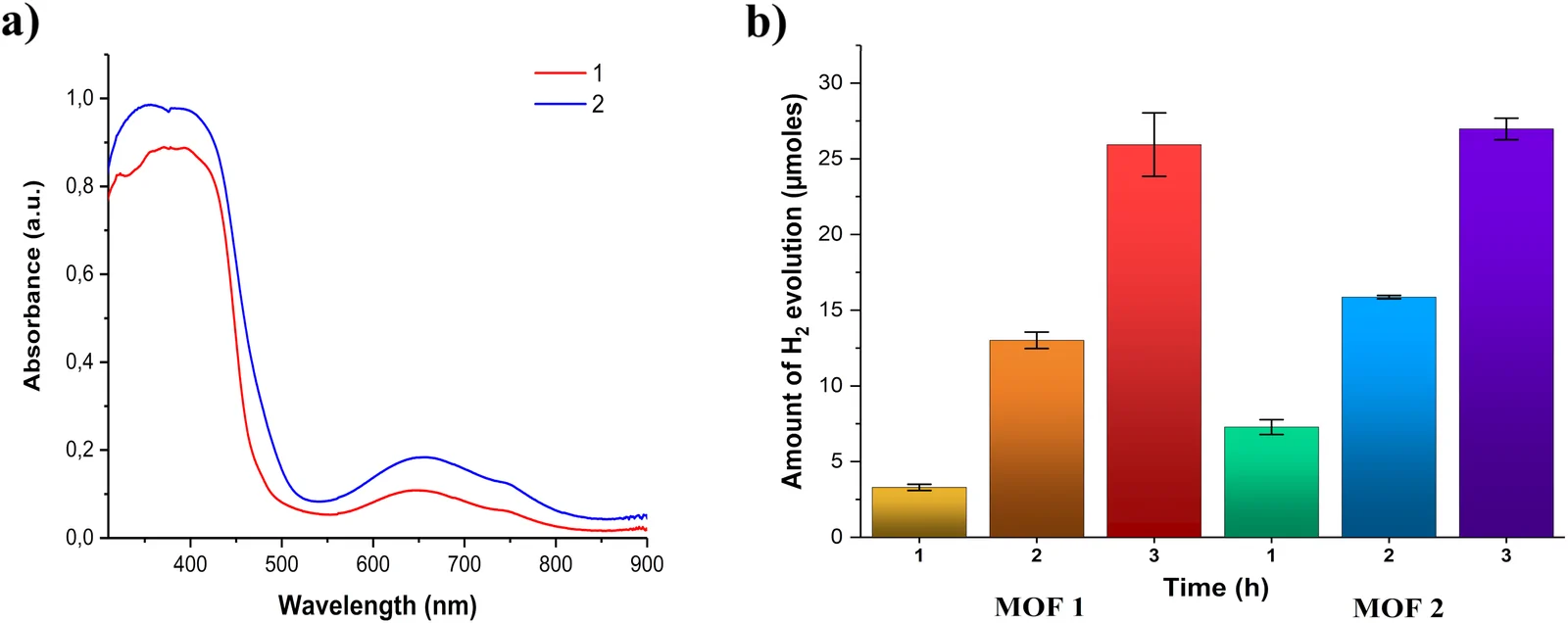
(a) Solid state absorption spectra of 1 and 2 from UV-vis-DRS measurements along a wavelength range of 300–900 nm. (b) Photocatalytic hydrogen production by 1 and 2 over 3 hours reaction. Reaction conditions: MOF photocatalyst (10 mg), H_2_O–MeOH solution (1 : 1, 10 mL), ascorbic acid (26 mg), LED AM 1.5G solar simulator light (100 mW cm^−2^, 380–1000 nm). Error bars represent the standard deviation between repetitions.

Encouraged by the physicochemical stabilities and favourable optical properties of the MOFs, we evaluated their performance as photocatalysts in photocatalytic hydrogen evolution reactions (HER). Using standardized AM 1.5G simulated sunlight irradiation in the wavelength range of 380–1000 nm and a combination of methanol and ascorbic acid as sacrificial reagents in water solutions, MOFs 1 and 2 exhibited photocatalytic activity in the HER reaction (Fig. 4b) with an average rate (including the induction period with lower H_2_ production rate) of 8.6 µmol h^−1^ and 9.0 µmol h^−1^, respectively.

No hydrogen was detected in the absence of a photocatalyst, confirming the photocatalytic activity of 1 and 2 (Fig. S7). It is worth noting that all tests were conducted without any cocatalysts and that in the case of MOF 1 the amorphous phase 1C acts as the active phase due to the phase transition occurring in water as mentioned above. Estimating the maximum production rates normalized by the mass of the photocatalyst used in these conditions, MOFs 1 and 2 showed rates of 1635 µmol g^−1^ h^−1^ and 1437 µmol g^−1^ h^−1^, respectively, surpassing some of the single-ligand TBAPY-based MOFs reported in the literature^8,39^ (Table S7). Although these kinds of mass-normalized values are commonly reported in the literature,^2,3,5^ it is important to note that they are typically extrapolated estimate values and scaling up does not necessarily result in a linear increase in hydrogen production rates, particularly without further optimization.^40,41^

To further evaluate reusability, long-term stability and potential variations in hydrogen production rates, both MOFs were also tested over an extended period spanning three consecutive reaction cycles under identical reaction conditions (Fig. 5). In the case of MOF 1, a significant decrease in hydrogen production is observed during the second and third reaction cycles (Fig. 5a). PXRD analysis (Fig. 5c) revealed that MOF 1 could not be regenerated after three cycles using the procedure described above, owing to its partial degradation into its components. The TBAPY ligand appears to form a hydrogen bonded framework (HOF) structure corresponding to the structure with CSD identifier MUDSIQ, which has also been reported to exhibit photocatalytic activity by Aitchison *et al.*^42^ However, after the first cycle where the hydrogen production rate reaches its maximum, the MOF 1 structure can still be regenerated (Fig. S18) with a similar irregular block-like morphology as the pristine 1 (Fig. S22), although a slight decrease in average crystal size is observed. Together these observations indicate somewhat short-lived stability of 1 in these cyclic processes or reaction conditions. It is assumed that prolonged exposure to water, combined with repeated drying of the powder between reaction cycles (as described above), may have led to the removal of structure-stabilizing solvent molecules and the disordered dangling DIA ligand. These effects could, in turn, have caused the complete collapse of the framework accompanied by hydrolysis of the TBAPY ligand carboxylate groups and framework decomposition. On the other hand, during the first reaction cycle the flexibility of 1 enabled both the formation of the amorphous phase 1C with possible missing linker defects, and the reversible transition back to crystalline 1 allowing the characterization of the preserved framework structure.

**Fig. 5:**
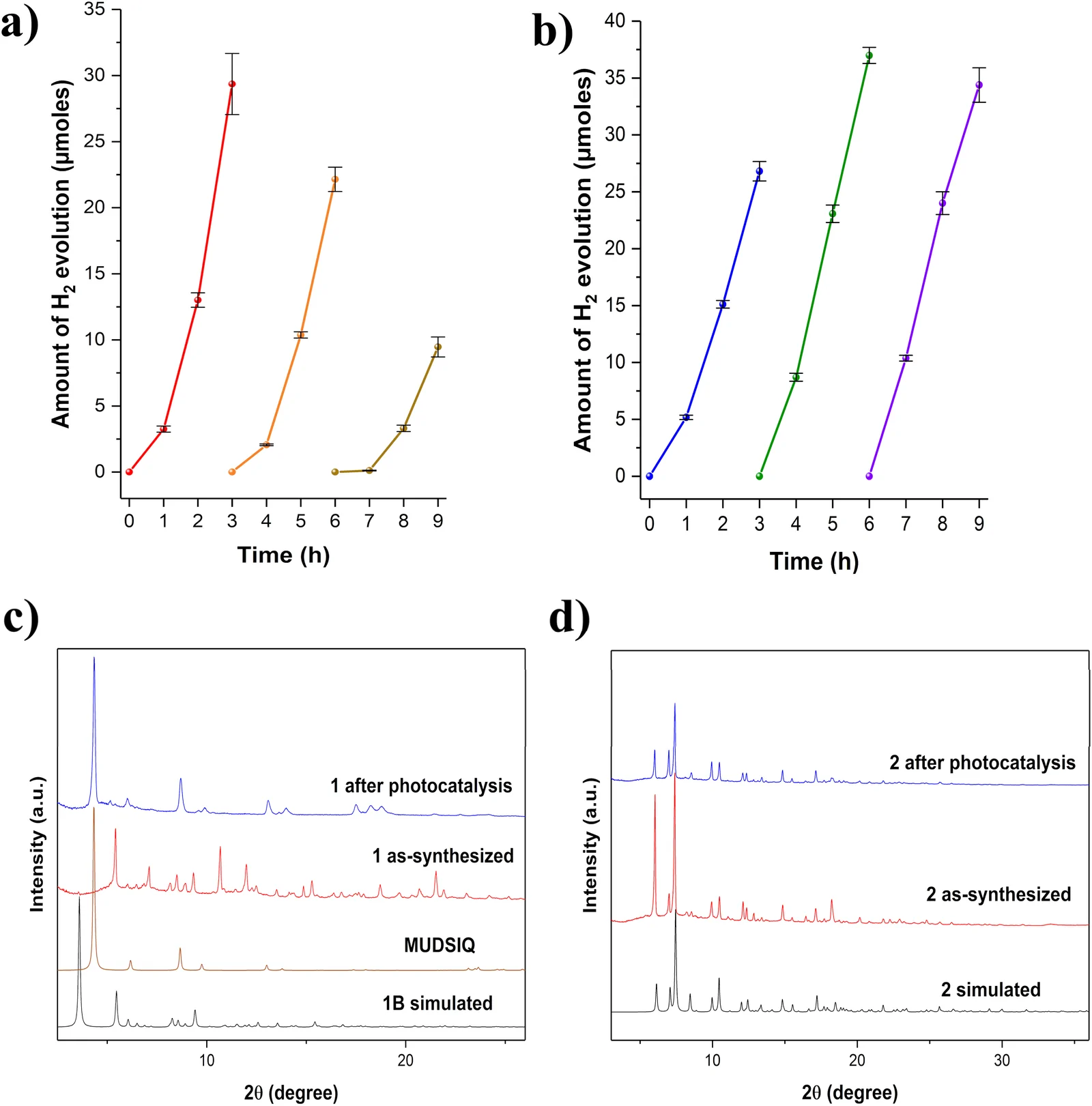
(a) Photocatalytic hydrogen production of 1 in three consecutive cycles. (b) Photocatalytic hydrogen production of 2 in three consecutive cycles. Reaction conditions: MOF photocatalyst (10 mg), H_2_O–MeOH solution (1 : 1, 10 mL), ascorbic acid (26 mg), LED AM 1.5G solar simulator light (100 mW cm^−2^, 380–1000 nm). Error bars represent the standard deviation between repetitions. (c) PXRD comparison of 1 before and after recycling test (and regeneration of 1 with previously mentioned procedure). (d) PXRD comparison of 2 before and after recycling test.

In contrast, MOF 2 demonstrates superior performance compared to MOF 1, as evidenced by its nearly identical hydrogen production rates maintained over three consecutive cycles (Fig. 5b) and by the preservation of its structural integrity under these reaction and processing conditions (Fig. 5d). Additionally, based on SEM analysis, 2 did not display any significant changes in the irregular block-like particle and crystal morphologies after the reaction (Fig. S22). These observations motivated us to study the performance of MOF 2 further by determining its apparent quantum efficiency (AQE) for photocatalytic hydrogen production (see the SI for details). Under 450 nm ± 12 nm irradiation the maximum estimated AQE value of MOF 2 reaches 2.16% while the average value (including the slower rate induction period) was 1.40%. The values of this level are relatively high since values above 1% are quite rare among MOF photocatalysts reported in the literature^2,3,5,43^ especially when considering the lack of need for any cocatalysts or photosensitizers with MOF 2. The AQE of MOF 2 surpasses the values of similar TBAPY single-ligand Sc- and Ti-based MOFs^8,10^ although remaining somewhat lower than the values achieved by certain Ni-based MOFs^7,9^ (Table S7). This difference may arise from the less efficient light absorption of the secondary DIP ligand and/or less efficient particle morphology. The proposed photocatalytic mechanism of MOF 2 is expected to proceed *via* the common ligand-to-metal charge transfer (LMCT) process, where upon photoexcitation the excited electrons transfer from the ligands to the nickel nodes which act as active proton reduction sites for generating hydrogen while the photogenerated holes are consumed by the sacrificial reagents, as similarly concluded in previous studies of several TBAPY single-ligand MOFs.^7,9,10,39^ It is worth noting, however, that direct comparisons between photocatalysts remain challenging because photocatalytic performance is strongly influenced by experimental factors, such as reactor design, photocatalyst loading, and light distribution within suspended catalyst particles.^40,41^

Nevertheless, the excellent stability of MOF 2 and its successful application in photocatalytic HER with an AQE comparable to or exceeding that of several related MOFs, demonstrate the potential of the mixed-ligand strategy for the design of efficient and practically applicable MOF photocatalysts. The superior performance of 2 relative to 1, is most likely attributable to its more rigid and seemingly defect-free framework, arising from improved size matching between the DIP and TBAPY ligands. This results in a denser structure exhibiting stronger non-covalent interactions and therefore a more stable framework. Some studies have suggested a relationship between decreased water stability and the presence of defects in MOF structures^44,45^ whereas others have reported enhanced photocatalytic performance in defect-engineered MOF structures.^46^ Therefore, while MOF 1 shows a slight improvement in the maximum photocatalytic hydrogen production rate (1635 µmol g^−1^ h^−1^*vs.* 1437 µmol g^−1^ h^−1^ of MOF 2) possibly owing to the generation of missing linker defects associated with its flexible and reversible structural transformation or its increased specific surface area, the superior long-term activity and stability of MOF 2 ultimately establish it as a more promising candidate for photocatalytic applications.

## Conclusions

The development of efficient photocatalysts for photocatalytic hydrogen production or overall water splitting is widely recognized as a challenging task, but at the same time it remains worthwhile and highly promising direction of research. In this study we present two novel MOF structures of 1 (Ni-DIA-TBAPY) and 2 (Ni-DIP-TBAPY) designed for photocatalytic applications. Structural characterization revealed that both MOFs contain practically identical secondary building units, yet adopt markedly different 3D-frameworks and pore structures. The dangling linker defect-containing 1 demonstrated more porous and flexible structure, as evidenced by reversible crystalline-to-amorphous transition induced by solvent exchange or thermal activation, whereas the denser structure 2 displayed a more rigid structural behaviour, remaining crystalline even upon boiling in water. With very similar optical properties, both MOFs demonstrated photocatalytic hydrogen production from water under AM 1.5G simulated solar irradiation in the presence of sacrificial reagents, but without the need for any noble metal cocatalysts. While 1 partially degraded under prolonged processing and reaction conditions, leading to decrease in its photocatalytic activity, 2 in contrast, showed no significant loss in photocatalytic hydrogen production rate or evidence of structural degradation. Overall, this work presents two new MOF structures that provide additional experimental insight into the design of stable MOFs and contribute to the ongoing development of novel noble-metal-free photocatalysts, advancing efforts toward more economical and efficient photocatalysts.

## Conflicts of interest

There are no conflicts to declare.

## Supplementary Material

RA-016-D6RA08963A-s001

RA-016-D6RA08963A-s002

## Data Availability

CCDC 2539232–2539235 contain the supplementary crystallographic data for this paper.^47*a*–*d*^ The data supporting this article have been included as part of the supplementary information (SI). Supplementary information: experimental NMR spectra, additional crystallographic data, additional PXRD analyses, TGA-curves, N_2_-adsorption isotherms, gas chromatography analysis details, optical band gap estimates, SEM analyses. See DOI: https://doi.org/10.1039/d6ra08963a.
