# Supplementary material for: Development and comparative investigation of structural and functional properties of Ni-based metal–organic frameworks designed for photocatalytic hydrogen production
Source: RSC Adv. 2026 Sep 28. Online ahead of print. doi: 10.1039/d6ra08963a (PMC13618343; doi:10.1039/d6ra08963a)
Supplement: Supplementary file 1 [file RA-OLF-D6RA08963A-s001.pdf]

Supplementary Information (SI) for RSC Advances.
This journal is © The Royal Society of Chemistry 2026
Supplementary Information (SI) for RSC Advances.
This journal is © The Royal Society of Chemistry

Supplementary information

Development and comparative investigation of structural and functional properties of Ni-based metal-organic frameworks designed for photocatalytic hydrogen production

*Antti Marttinen, Manu Lahtinen**

Department of Chemistry, University of Jyväskylä, Jyväskylä, Finland.

Contents

NMR measurements ... 2

Crystallographic structure analysis of DIP ligand ... 3

Powder x-ray diffraction (PXRD) analyses of the synthesized ligands ... 5

Gas chromatography analyses ... 6

Apparent quantum efficiency (AQE) measurement and calculation details ... 7

Additional crystallographic analysis and data of the MOF single crystals ... 9

Additional powder x-ray diffraction analyses of the synthesized MOFs ... 13

Thermogravimetric analyses and N_2 adsorption measurements ... 18

Tauc plots of the synthesized MOFs derived from UV-VIS-DRS data ... 19

Scanning electron microscopy (SEM) analysis of MOF morphologies ... 20

References ... 22

NMR measurements

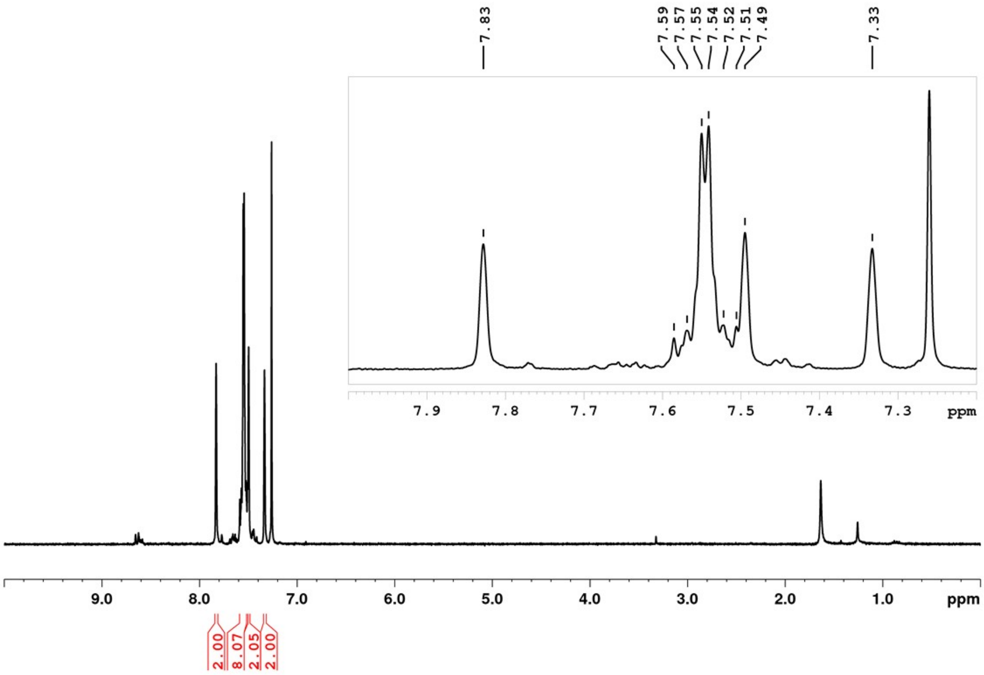

Fig. S1. ^{1}H NMR spectrum of DIA ligand in $CDCl_3$ (residual $CHCl_3$ peak at 7.26 ppm, impurity H_2O peak at 1.63 ppm). The inset shows the zoomed-in aromatic region of the spectrum.

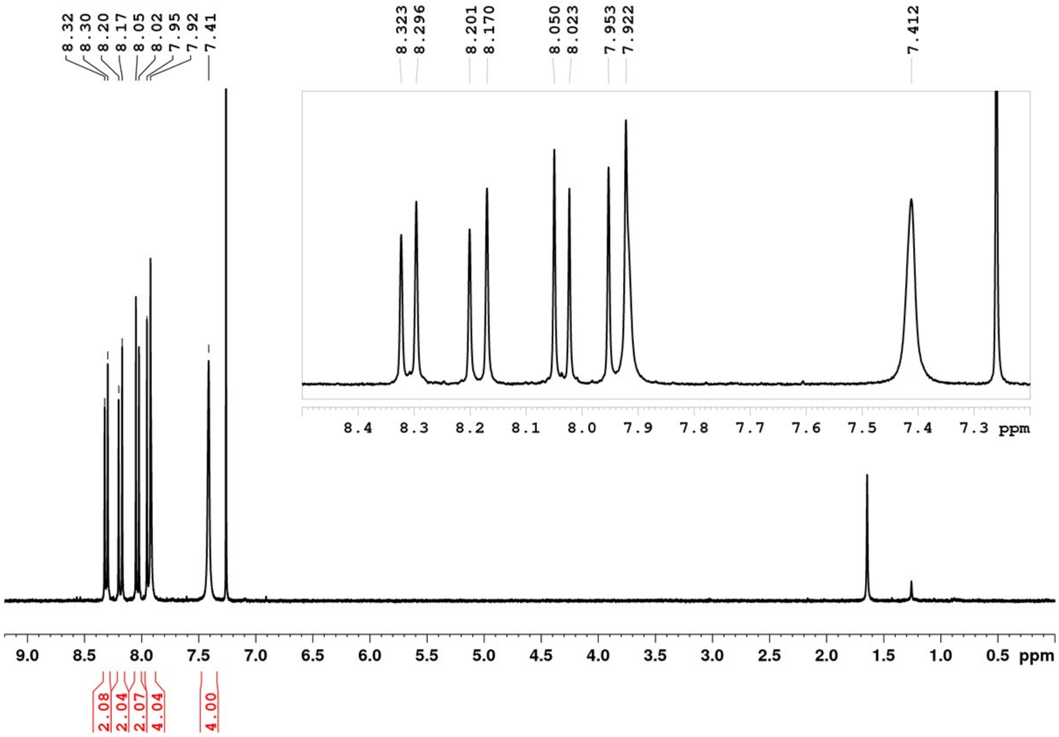

Fig. S2. ^{1}H NMR spectrum of DIP ligand in $CDCl_3$ (residual $CHCl_3$ peak at 7.26 ppm, impurity H_2O peak at 1.64 ppm). The inset shows the zoomed-in aromatic region of the spectrum.

Crystallographic structure analysis of DIP ligand

The as-synthesized 1,6-diimidazolepyrene (DIP) was crystallized by slow evaporation from $CHCl_3$ solution. Colourless block crystal was measured using Rigaku XtaLAB Synergy-R single source high flux rotating anode X-ray diffractometer (as described in the experimental section). The ligand crystallizes in monoclinic space group of $P2_1/n$ (no. 14) and due to its symmetry only half of the ligand is present in the asymmetric unit (Fig.S3a). No solvents are present in the structure and the individual molecules pack with the imidazole N-atoms being closest to neighbouring imidazole H-atoms (Fig.S3b), but not close enough to form strong or moderate hydrogen bonds (very weak bonds with D-A length = 3.44 Å, angle = 140°). The π-π stacking interactions with neighbouring molecules are also very weak with closest centroid-centroid distances being 4.04 Å (Fig.S3c). Crystallographic data of the structure solution is presented in table S1.

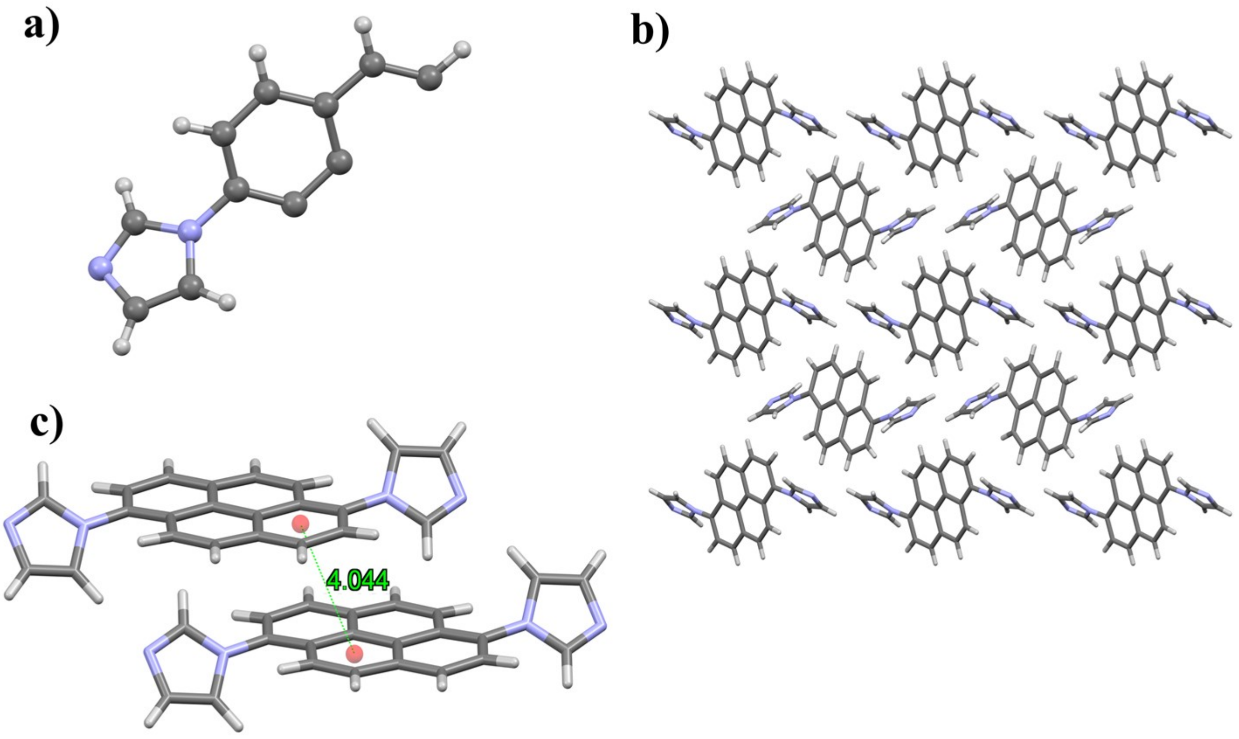

Fig. S3. a) Asymmetric unit of the DIP ligand. b) Packing of the DIP ligand along a-direction. c) Weak π-π stacking interaction between neighbouring ligands. Atom colour codes N: blue, C: grey, H: white.

Table S1: Crystallographic data of DIP ligand.

CCDC deposition number	2539232
Empirical formula	$C_{22}H_{14}N_4$
Formula weight	334.37
Temperature/K	120.01(10)
Crystal system	monoclinic
Space group	$P2_1/n$
a/Å	5.43930(10)
b/Å	11.1353(2)
c/Å	12.7717(2)
α/°	90
β/°	95.565(2)
γ/°	90
Volume/Å^3	769.91(2)
Z	2
ρ_{calc}g/cm^3	1.442
μ/mm^{-1}	0.696
F(000)	348.0
Crystal size/mm^3	0.26 × 0.12 × 0.1
Radiation	Cu Kα (λ = 1.54184)
2Θ range for data collection/°	10.562 to 157.83
Index ranges	$-6 \le h \le 6$, $-14 \le k \le 14$, $-15 \le l \le 16$
Reflections collected	16074
Independent reflections	1650 [R_{int} = 0.0475, R_{sigma} = 0.0209]
Data/restraints/parameters	1650/0/146
Goodness-of-fit on F^2	1.070
Final R indexes [I>=2σ (I)]	R_1 = 0.0476, wR_2 = 0.1239
Final R indexes [all data]	R_1 = 0.0498, wR_2 = 0.1256
Largest diff. peak/hole / e Å^{-3}	0.89/-0.19

Powder x-ray diffraction (PXRD) analyses of the synthesized ligands

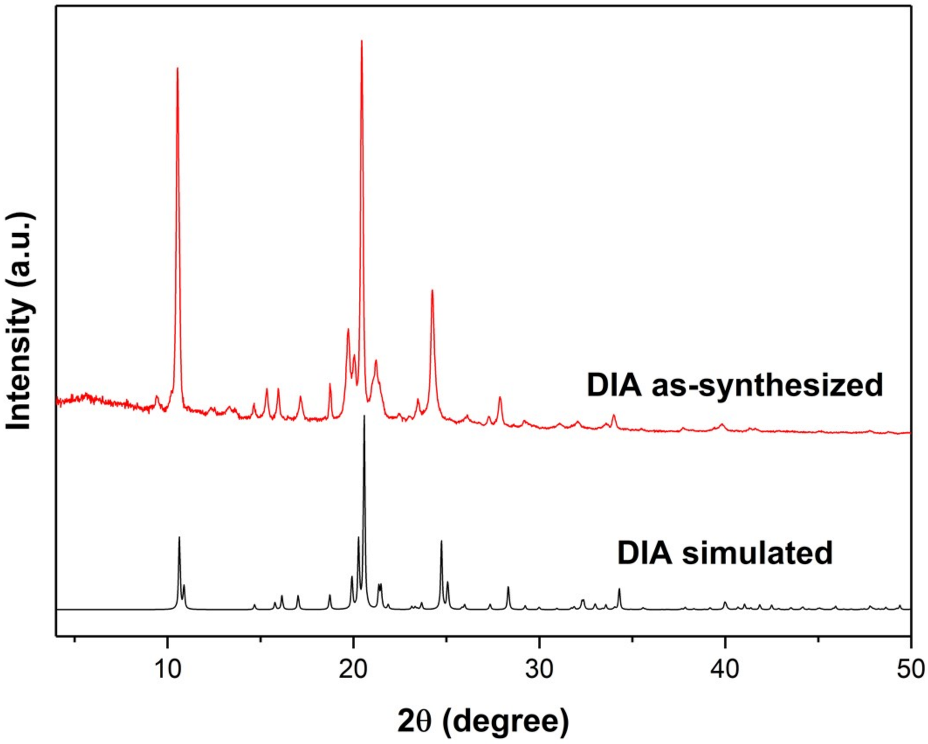

Fig. S4. PXRD-pattern of synthesized DIA ligand compared to SCXRD simulated pattern (retrieved from CSD with refcode ETAWIG).

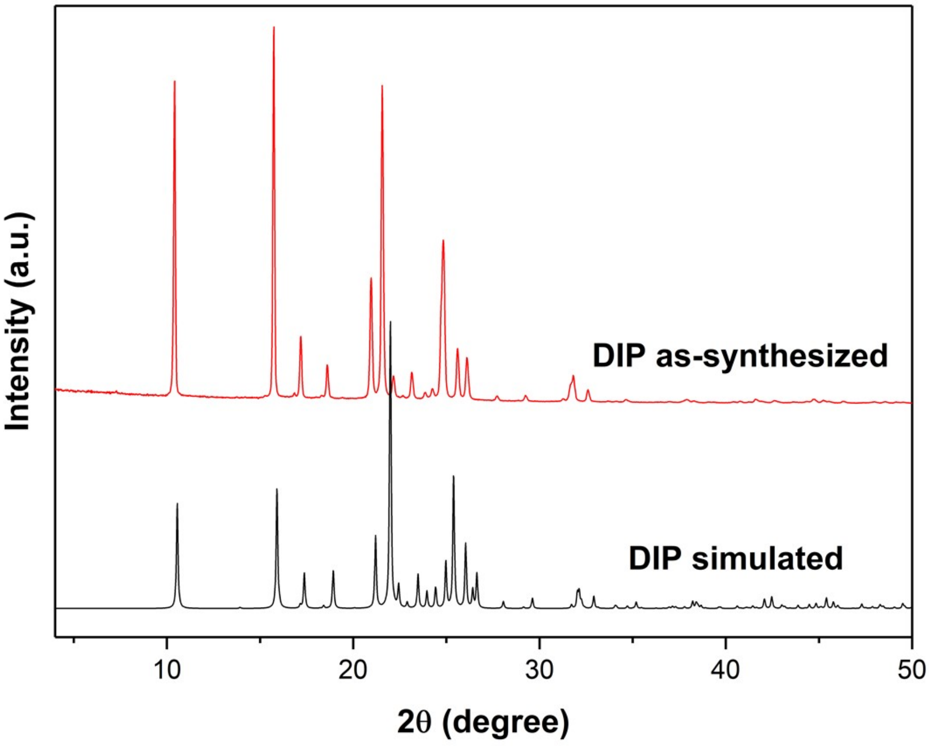

Fig. S5. PXRD-pattern of synthesized DIP ligand compared to SCXRD simulated pattern (reported in this study).

Gas chromatography analyses

Gas chromatograph analyses were conducted with an Agilent 8860 GC system of which details are listed in table S2.

Table S2: Agilent 8860 GC system conditions.

Carrier gas	Argon (99.999 %)
Column	HP-PLOT Molesieve 5Å (15 m length, 0.32 mm ID)
Column/oven temperature	50 °C (isothermal)
Column flow rate	1.0 ml/min
TCD temperature	200 °C
TCD makeup gas flow rate	5 ml/min
TCD reference gas flow rate	22 ml/min
S/SL inlet temperature	150 °C
Split ratio	20:1

Hydrogen calibration curve (Fig. S6) was prepared by injecting specific amounts of pure hydrogen gas with a gas-tight syringe (Agilent) into the reaction vial with 10 ml of H_2O and 13 ml argon purged headspace inside the reaction vial. Before sampling, the gas-tight syringe was purged with argon and 200 µl of argon was injected into the headspace. After 1 minute of settling, 200 µl sample was withdrawn and injected into the GC and at least triplicate samples were measured for each amount of hydrogen injected. This sampling method was used to maintain standard pressure and prevent formation of a partial vacuum inside the headspace after several withdrawn samples. Identical sampling method was used to analyze the evolved hydrogen in the photocatalytic reactions with at least triplicate samples at each point of time to minimize errors.

Apparent quantum efficiency (AQE) measurement and calculation details

Apparent quantum efficiency (AQE) measurements were conducted similarly as the photocatalytic hydrogen production experiments detailed in the main text, but instead of simulated solar irradiation, 450 nm±12 nm LED light was used for irradiation. The irradiance value was measured with a optical power meter (Thorlabs, PM-100D with a S401C sensor) which was converted to a number of $7.296 \cdot 10^{20}$ (1211.49 µmoles in molar amount) incident photons per solution surface area (5.04 cm^2) per hour. The maximum hourly H_2 production for MOF **2** at 450 nm irradiation was 13.11 µmoles and the total H_2 production during the 3-hour reaction (including slower rate induction period) was 25.45 µmoles determined by GC. AQE values were then calculated with the equation below, where the coefficient of 2 is present due to the reaction stoichiometry.

$$AQE\ (\%) = \frac{2 \times n_{H_2\,produced}}{n_{incident\ photons}} \times 100\ \%$$

$$AQE_{max} = \frac{2 \times 13.11\ \mu moles}{1211.49\ \mu moles} \times 100\ \% = 2.164\ \%$$

$$AQE_{avg} = \frac{2 \times 25.45\ \mu moles}{3634.47\ \mu moles} \times 100\ \% = 1.400\ \%$$

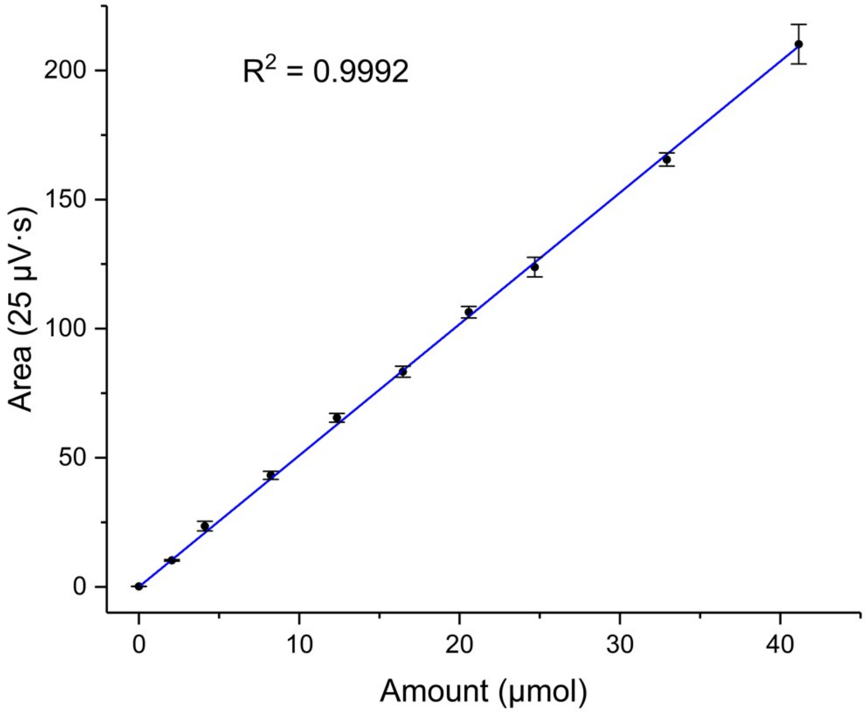

Fig. S6. Calibration curve of hydrogen amount in micromoles. Error bars show the standard deviation between measurement repetitions.

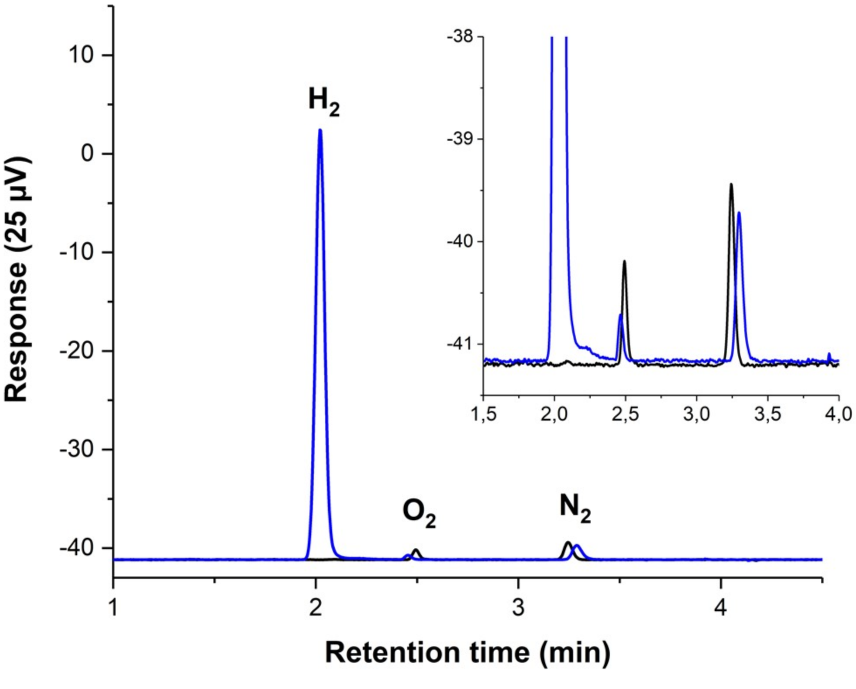

Fig. S7. Example chromatogram (blue) of the 3-hour photocatalytic HER-reaction test for **1** compared to a chromatogram (black) of the 3-hour photocatalytic test in the absence of photocatalyst (H_2O:MeOH:ascorbic acid solution). Inset shows the zoomed-in region of the peaks. Minor peaks of oxygen and nitrogen are due to small presence of air in the needle of the gas-tight syringe.

Additional crystallographic analysis and data of the MOF single crystals

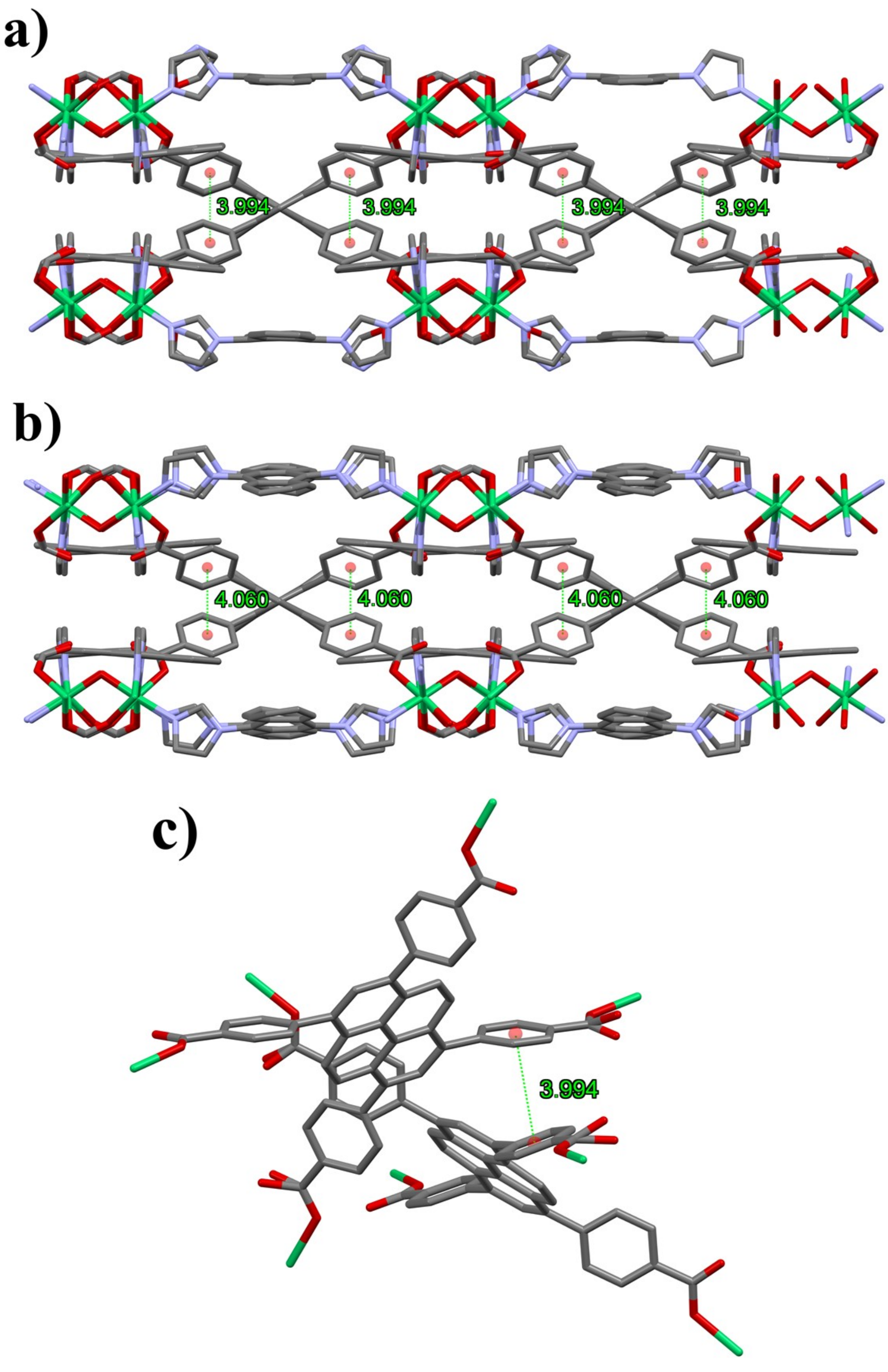

Fig. S8. Partial structure views of **1**: a) Weak π-π stacking interactions of **1** along the *a*-axis b) Weak π-π interactions of **1B** along the *a*-axis. c) Same interaction of **1** but including only the ligands in question. π-π interactions presented as light green lines, with centroid-centroid distances reported in Å. Atom colour codes Ni: green, N: blue, O: red, C: grey.

a)

b)

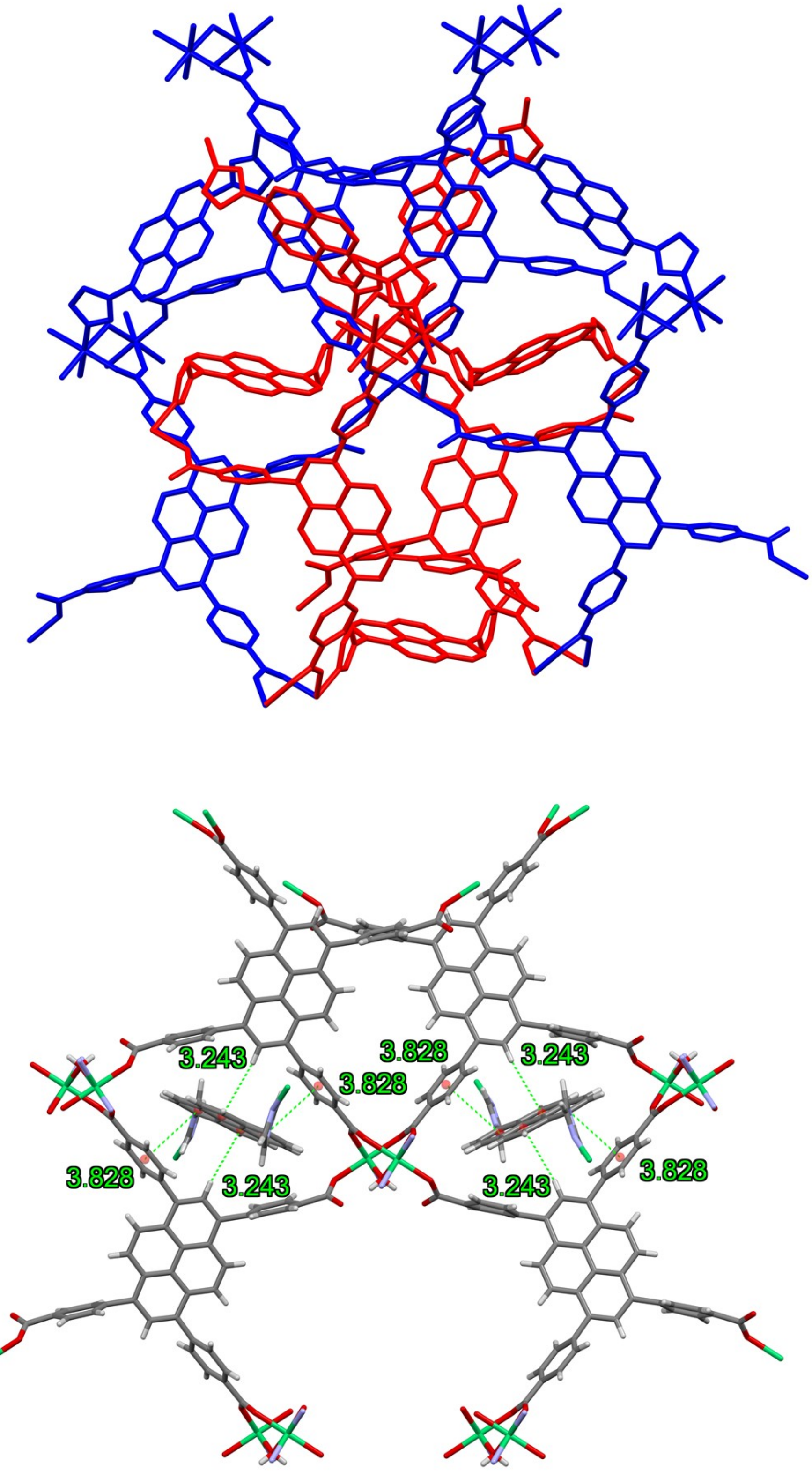

Fig. S9. Partial structure views of **2**: a) self-penetration/-catenation of the framework of **2** shown by blue and red colored parts b) Weak π-π stacking and C-H-π interactions of **2**. π-π/C-H-π interactions presented as light green lines, with centroid-centroid/H-π distances reported in Å. Atom colour codes Ni: green, N: blue, O: red, C: grey, H: white.

Table S3: Crystallographic data of MOF structures **1**, **1B** and **2**.

**Structure**	**1**	**1B**	**2**
CCDC deposition number	2539233	2539234	2539235
Empirical formula	$C_{199.5}H_{205.7}N_{24.9}Ni_4O_{41.4}$	$C_{160}H_{123.2}N_{14.4}Ni_4O_{30.4}$	$C_{96}H_{74}N_{10}Ni_2O_{14}$
Formula weight	3849.42	2968.76	1709.07
Temperature/K	120.00(10)	273.01(10)	120.00(18)
Crystal system	orthorhombic	orthorhombic	monoclinic
Space group	Ibam	Ibam	C2/c
a/Å	29.38740(10)	29.1893(2)	14.1650(2)
b/Å	31.92620(10)	32.3287(3)	28.8102(4)
c/Å	48.4754(2)	49.0640(4)	21.3532(3)
α/°	90	90	90
β/°	90	90	101.8640(10)
γ/°	90	90	90
Volume/Å^3	45481.0(3)	46299.4(7)	8528.0(2)
Z	8	8	4
ρ_{calc}g/cm^3	1.124	0.852	1.331
μ/mm^{-1}	0.950	0.782	1.134
F(000)	16162.0	12314.0	3552.0
Crystal size/mm^3	0.17 × 0.13 × 0.13	0.15 × 0.14 × 0.12	0.14 × 0.1 × 0.08
Radiation	Cu Kα (λ = 1.54184)	Cu Kα (λ = 1.54184)	Cu Kα (λ = 1.54184)
2Θ range for data collection/°	5.478 to 158.54	5.442 to 158.344	7.076 to 158.426
Index ranges	-37 ≤ h ≤ 37, -39 ≤ k ≤ 40, -61 ≤ l ≤ 58	-36 ≤ h ≤ 36, -38 ≤ k ≤ 40, -51 ≤ l ≤ 62	-17 ≤ h ≤ 17, -36 ≤ k ≤ 35, -26 ≤ l ≤ 27
Reflections collected	473897	151567	48336
Independent reflections	24784 [R_{int} = 0.0488, R_{sigma} = 0.0136]	25081 [R_{int} = 0.0616, R_{sigma} = 0.0334]	9139 [R_{int} = 0.0332, R_{sigma} = 0.0236]
Data/restraints/parameters	24784/93/967	25081/141/927	9139/12/607
Goodness-of-fit on F^2	1.033	1.041	1.051
Final R indexes [I>=2σ (I)]	R_1 = 0.0679, wR_2 = 0.2186	R_1 = 0.0758, wR_2 = 0.2264	R_1 = 0.0649, wR_2 = 0.1834
Final R indexes [all data]	R_1 = 0.0750, wR_2 = 0.2288	R_1 = 0.1061, wR_2 = 0.2529	R_1 = 0.0735, wR_2 = 0.1906
Largest diff. peak/hole / e Å^{-3}	0.74/-0.47	0.68/-0.49	0.94/-0.54

Table S4: Selected bond lengths (Å) and angles (°) of structure **1**.

Ni1-O39	2.0519(16)	Ni2-O96C	2.098(2)
Ni1-O92^1	2.0787(15)	Ni2-O64^1	2.0389(15)

Ni1-O40	2.0368(15)	O39-Ni1-O92^{1}	89.66(6)
Ni1-O65^{1}	2.0475(15)	O39-Ni1-N3	175.95(7)
Ni1-N15	2.0800(18)	O92^{1}-Ni1-N3	88.30(7)
Ni1-N3	2.075(2)	O40-Ni1-O39	93.32(6)
Ni2-O39	2.0478(15)	O65^{1}-Ni1-N3	85.19(7)
Ni2-O68	2.0657(15)	O39-Ni2-O96C	178.25(8)
Ni2-O42	2.0352(16)	O42-Ni2-N36^{1}	174.23(8)
Ni2-N36^{1}	2.0668(18)	O42-Ni2-O96C	85.29(8)

^{1}1/2+X,1/2-Y,+Z;

Table S5: Selected bond lengths (Å) and angles (°) of structure **1B**.

Ni1-O67^{1}	2.0510(18)	Ni2-O96B	2.094(2)
Ni1-O94^{2}	2.0775(16)	Ni1^{4}-O67	2.0382(16)
Ni1-O15	2.0381(16)	O67^{1}-Ni1-O94^{2}	90.19(8)
Ni1-O42^{1}	2.0462(17)	O94^{2}-Ni1-N3	86.86(9)
Ni1-N64^{3}	2.076(2)	O15-Ni1-O67^{1}	93.75(8)
Ni1-N3	2.078(2)	O42^{1}-Ni1-N3	85.66(10)
Ni2-O67	2.0469(17)	O67-Ni2-O17^{4}	93.81(8)
Ni2-O68	2.0384(16)	O67-Ni2-O96B	175.65(15)
Ni2-O41	2.0650(16)	O17^{4}-Ni2-N43	174.56(11)
Ni2-O17^{4}	2.0355(17)	O17^{4}-Ni2-O96B	84.74(15)
Ni2-N43	2.068(2)		

^{1}1/2+X,3/2-Y,1-Z; ^{2}1+X,+Y,1-Z; ^{3}+X,+Y,1-Z; ^{4}-1/2+X,3/2-Y,1-Z;

Table S6: Selected bond lengths (Å) and angles (°) of structure **2**.

Ni1-O41	2.0957(12)	O15-Ni1-O41	90.89(7)
Ni1-O15	2.0400(17)	O15-Ni1-O40^{1}	175.96(8)
Ni1-O40^{1}	2.0726(17)	O17^{2}-Ni1-N42	85.65(9)
Ni1-O17^{2}	2.0380(18)	N42-Ni1-O41	175.23(9)
Ni1-N2	2.076(2)	N42-Ni1-N2	94.45(9)
Ni1-N42	2.058(2)	O40^{1}-Ni1-O41	92.19(7)

^{1}-1+X,1-Y,-1/2+Z; ^{2}-X,+Y,1/2-Z;

Additional powder x-ray diffraction analyses of the synthesized MOFs

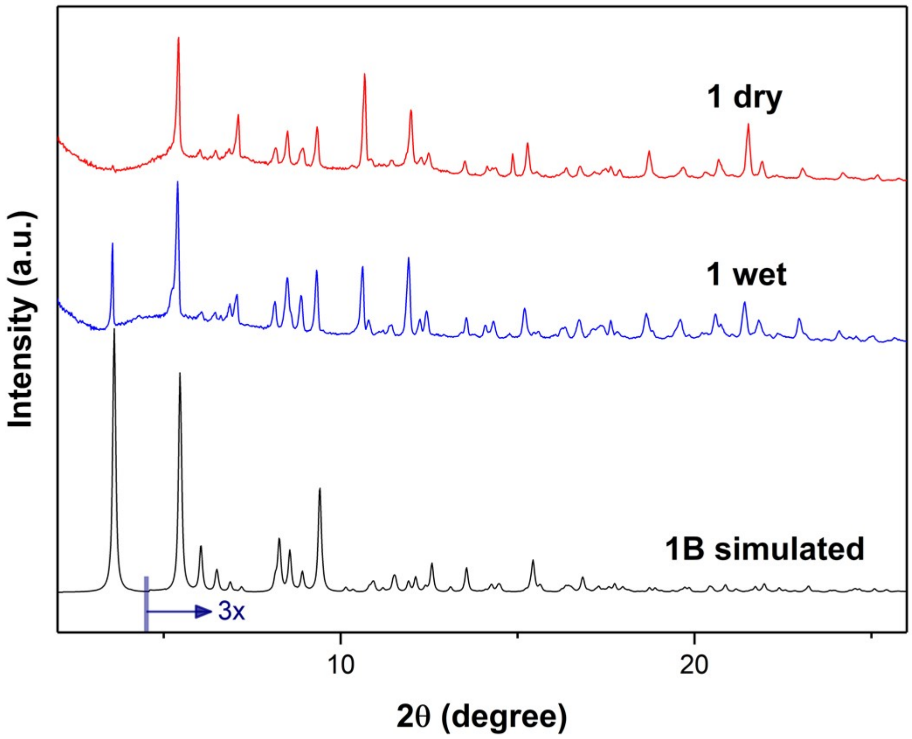

Fig. S10. PXRD-pattern of synthesized compound **1**: measured immediately after filtering while still wet/moist, and after drying, compared to the SCXRD simulated pattern of **1B**.

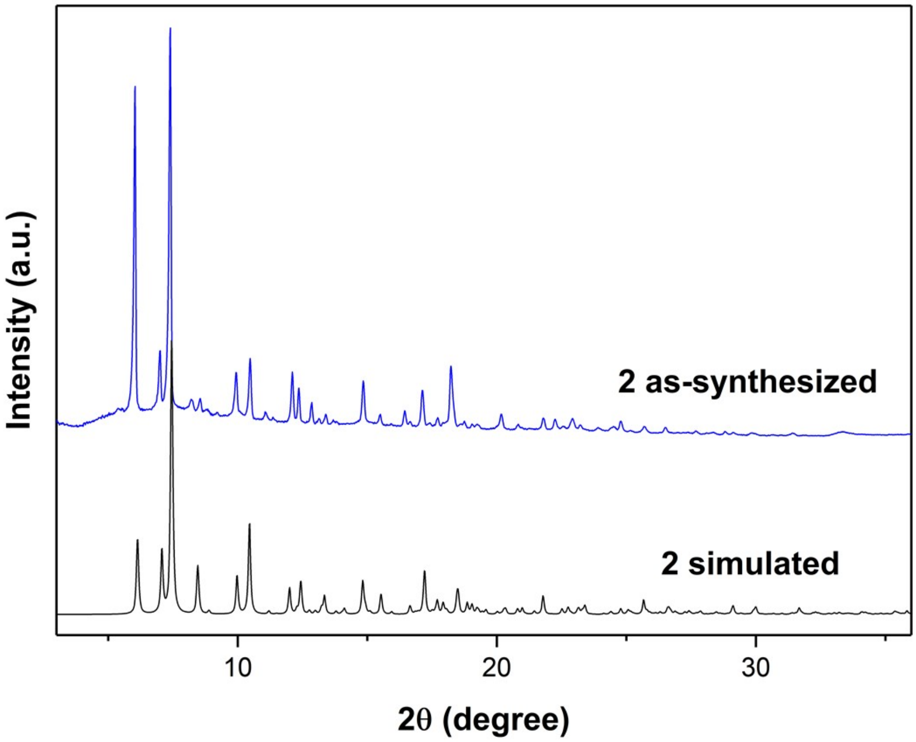

Fig. S11. PXRD-pattern of synthesized compound **2** compared to the SCXRD simulated pattern of **2**.

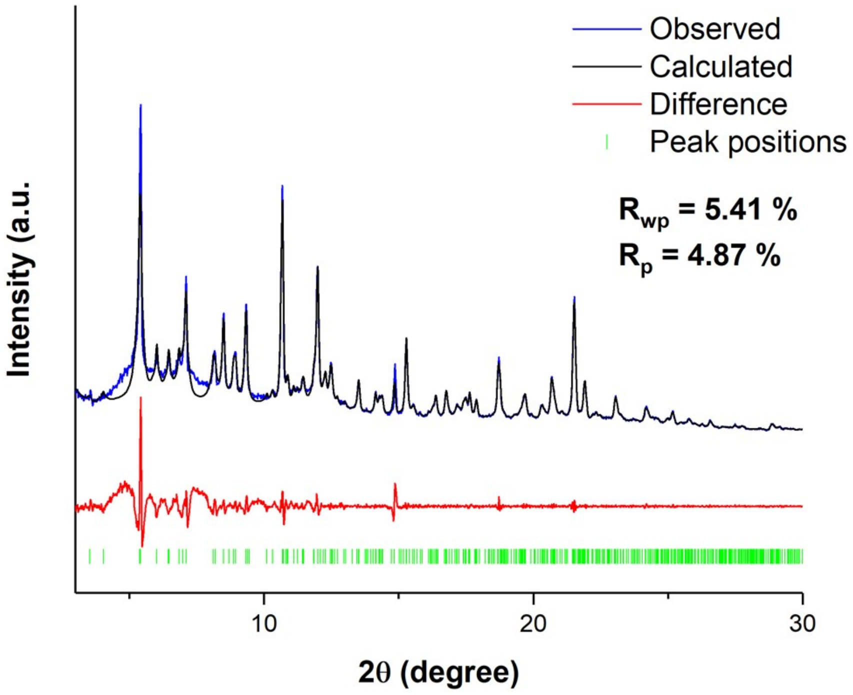

Fig. S12. Example of a Pawley refinement for MOF **1** showing the experimental PXRD pattern (blue), refined profile (black), difference plot (red) and characteristic Bragg peak positions (green).

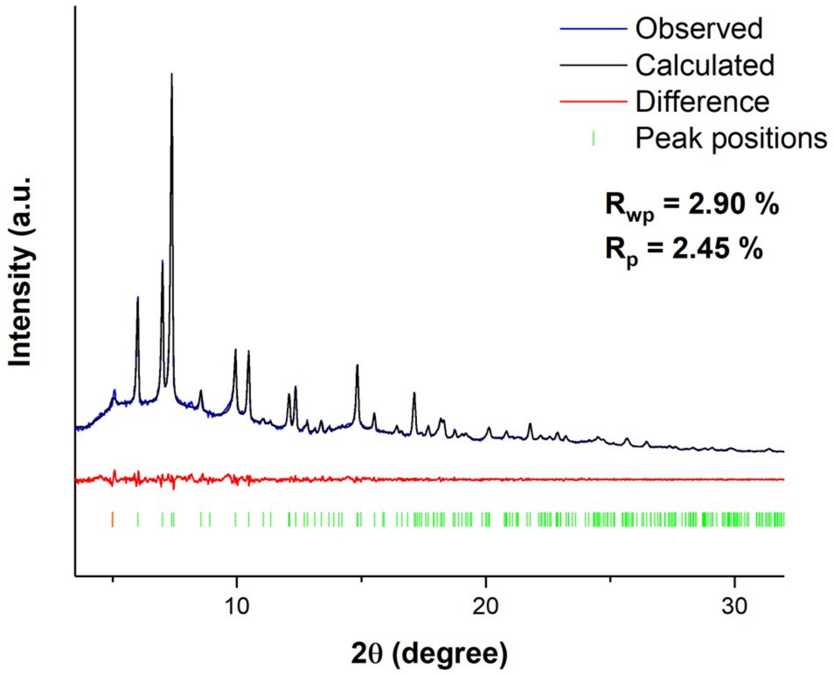

Fig. S13. Example of a Pawley refinement for MOF **2** showing the experimental PXRD pattern (blue), refined profile (black), difference plot (red) and characteristic Bragg peak positions (green). Single negligible secondary phase peak is marked in orange (observed only in some product batches).

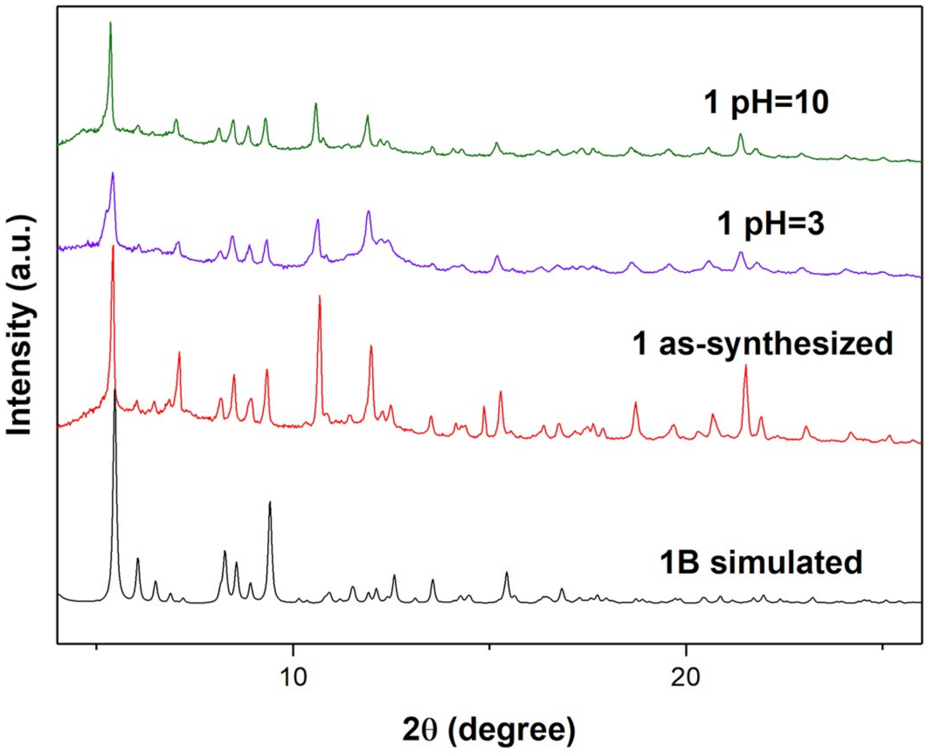

Fig. S14. PXRD-patterns of compound **1**: as-synthesized and the regenerated **1** after soaking in pH 3 (ascorbic acid) or pH 10 (sodium hydroxide) aqueous solutions, compared to the SCXRD simulated pattern of **1B**.

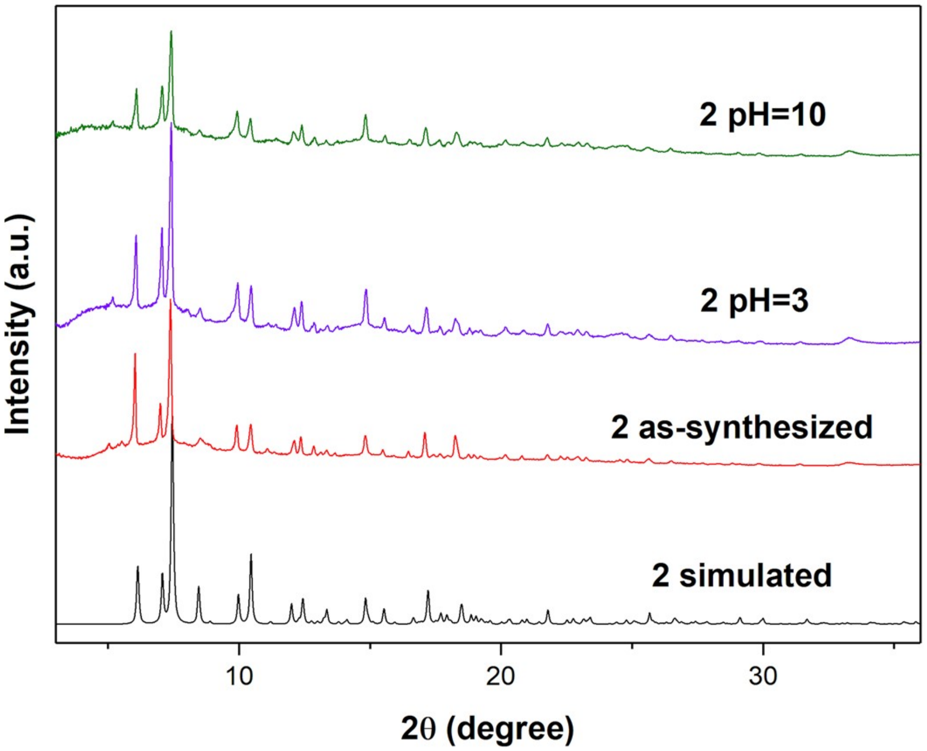

Fig. S15. PXRD-patterns of compound **2**: as-synthesized and after soaking in pH 3 (ascorbic acid) or pH 10 (sodium hydroxide) aqueous solutions, compared to the SCXRD simulated pattern of **2**.

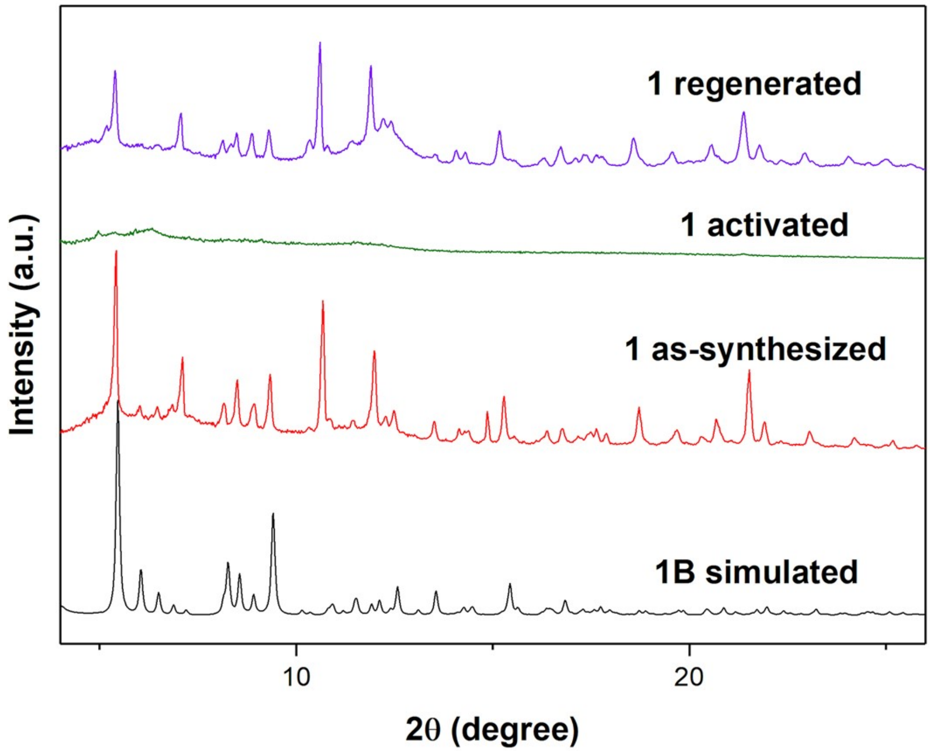

Fig. S16. PXRD-patterns of compound **1**: before and after activation under dynamic vacuum at 100 °C for 12 h along with the regenerated phase, compared to the SCXRD simulated pattern of **1B**.

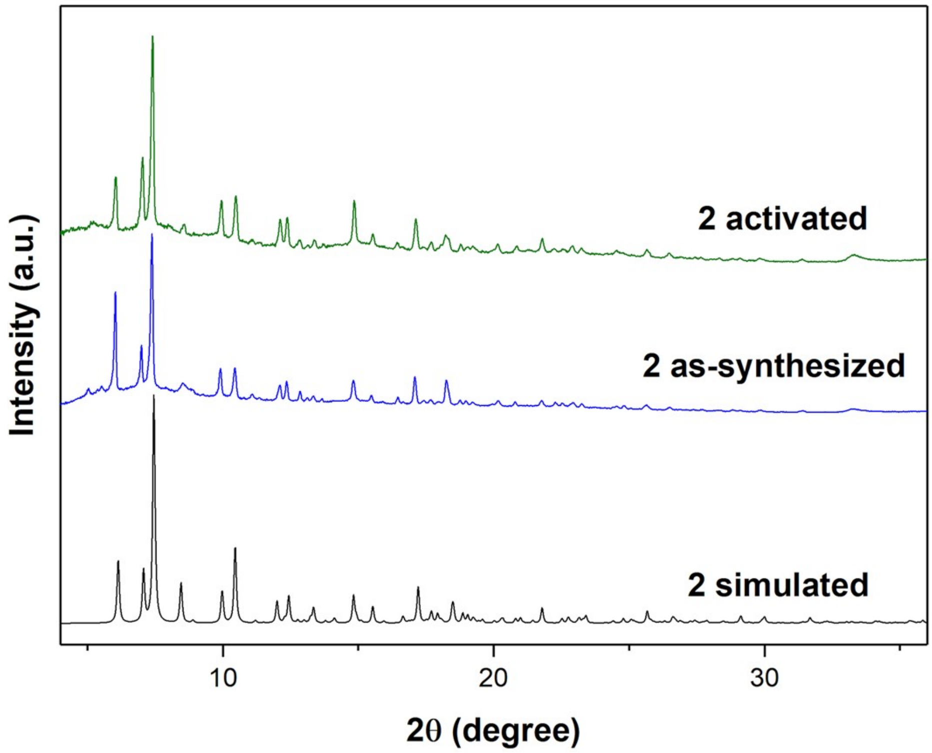

Fig. S17. PXRD-patterns of compound **2**: before and after activation under dynamic vacuum at 100 °C for 12 h, compared to the SCXRD simulated pattern of **2**.

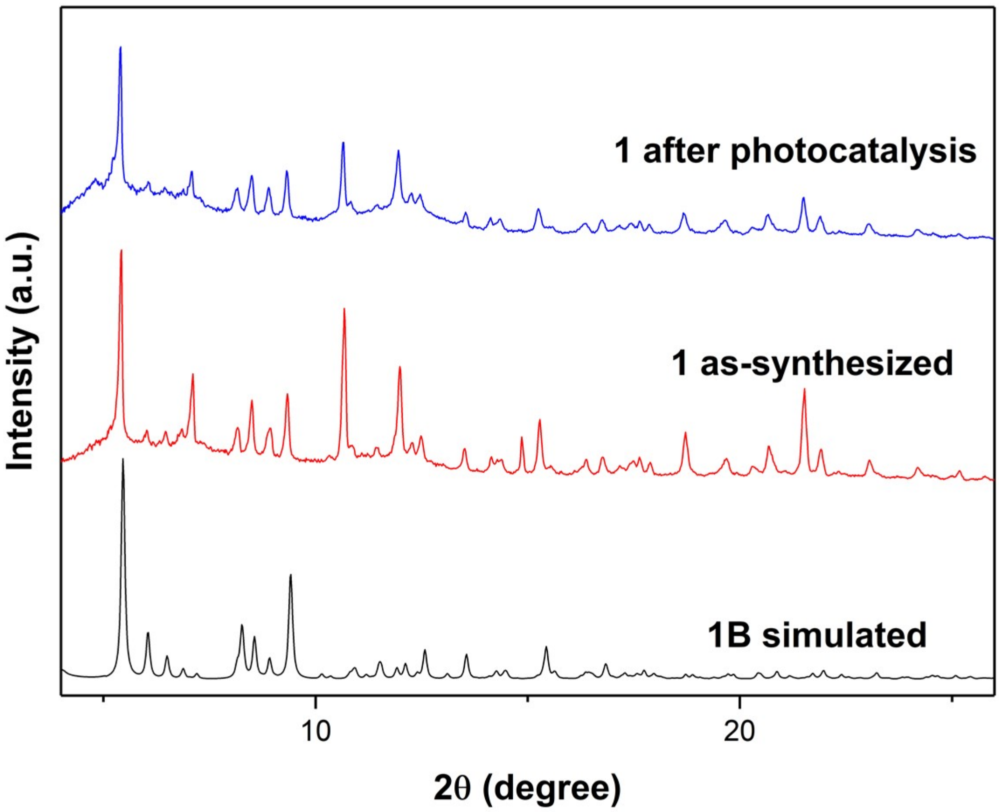

Fig. S18. PXRD-patterns of compound **1**: as-synthesized, and the regenerated **1** after the first 3-hour photocatalytic HER-reaction cycle, compared to the SCXRD simulated pattern of **1B**.

Thermogravimetric analyses and N_2 adsorption measurements

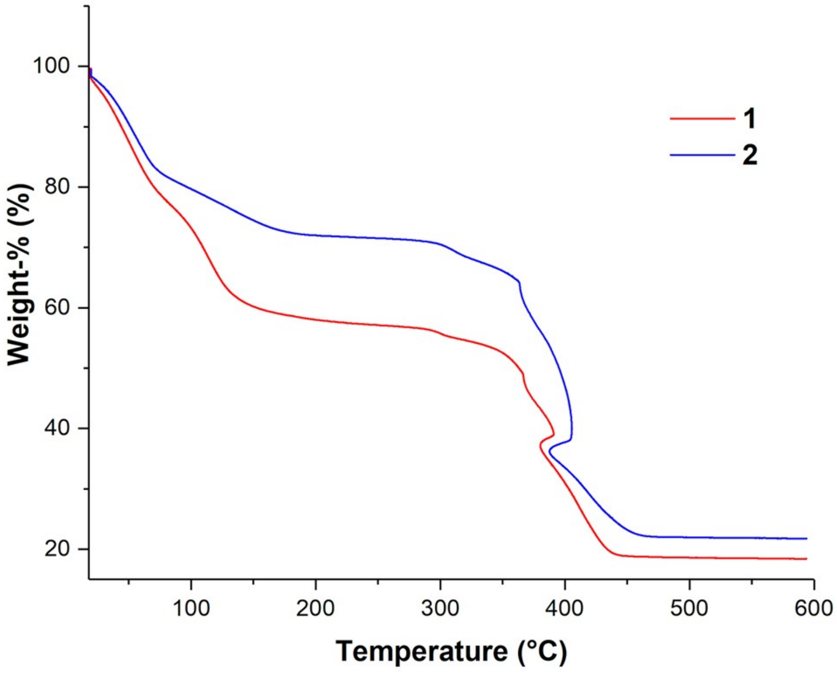

Fig. S19. TGA-curves of as-synthesized MOF **1** and **2**. Curves show a slight decrease in temperature in the range of 370-400 °C due to exothermic degradation processes. At given range sample temperature increases and subsequently decreases momentarily before reaching the programmed heating rate.

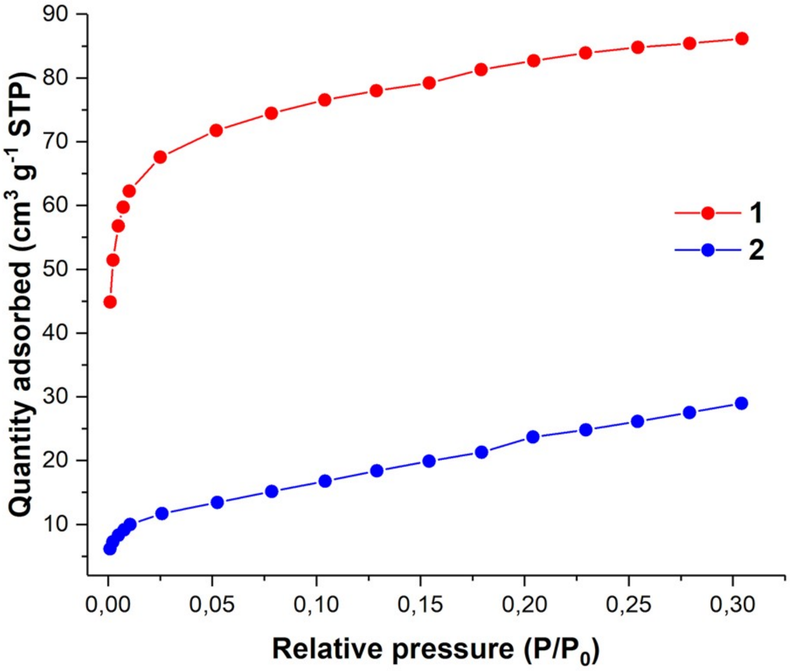

Fig. S20. N_2 adsorption isotherms (77 K) of as-synthesized MOF **1** and **2** after drying and degassing under dynamic vacuum at 100 °C for 12 h.

Tauc plots of the synthesized MOFs derived from UV-VIS-DRS data

a)

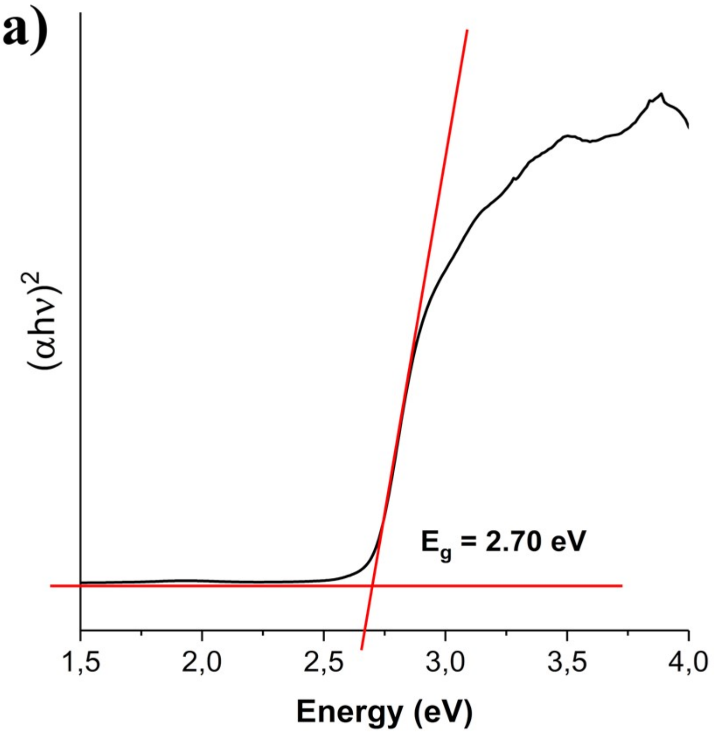

b)

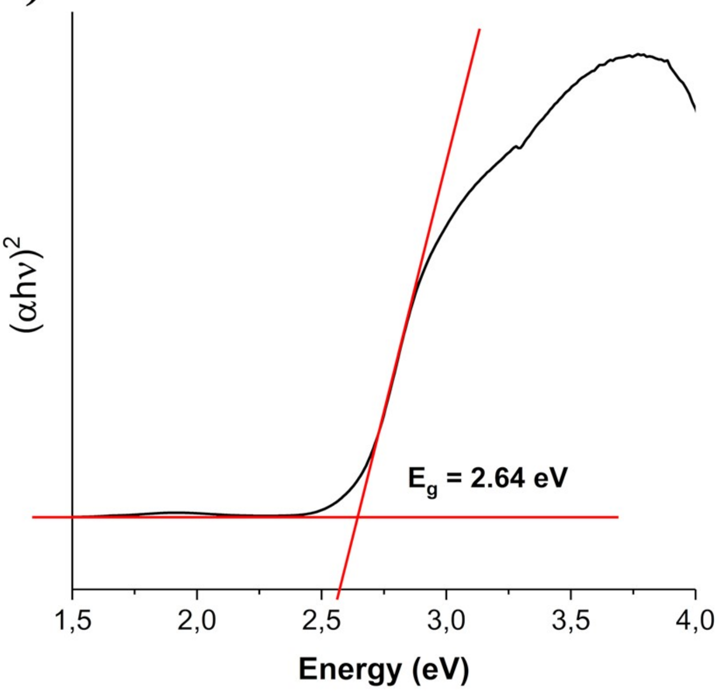

Fig. S21. Tauc-plots of **1** (a) and **2** (b) and their band gap estimates. α = absorption coefficient, h = Planck constant, v = light frequency and E_g = optical band gap.

Scanning electron microscopy (SEM) analysis of MOF morphologies

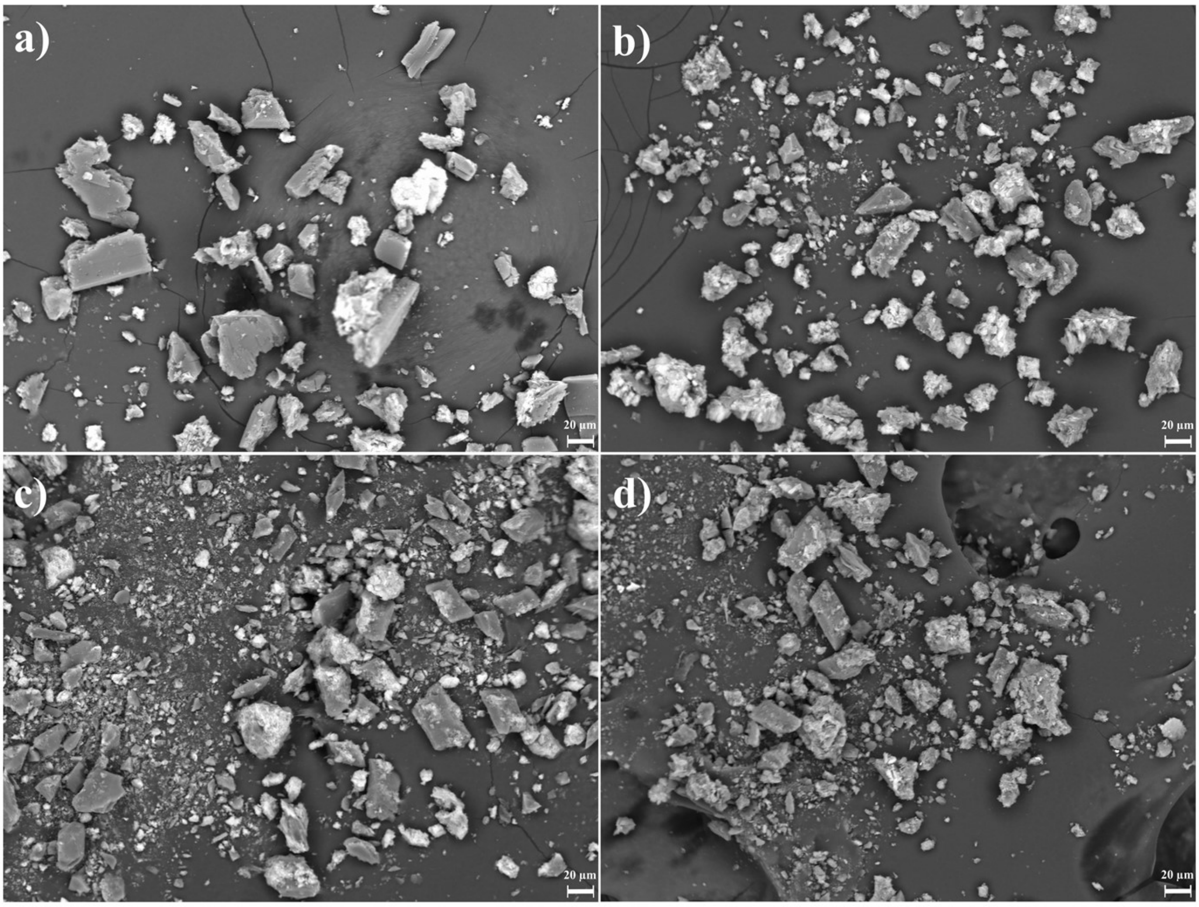

Fig. S22. SEM images at 800x magnification (Zeiss EVO-50, electron high tension voltage 20 kV, NTS BSD detector) of the bulk MOF products: a) as-synthesized MOF **1**. b) regenerated MOF **1** after the first 3-hour photocatalytic HER-reaction cycle. c) as-synthesized MOF **2**. d) MOF **2** after three photocatalytic HER reaction cycles.

Table S7: Comparison of the use of selected few similar TBAPY ligand-based photocatalysts in photocatalytic hydrogen evolution reactions.

**Photocatalyst**	**Light source**	**Sacrificial reagents**	**Co-catalyst**	**H_2 production rate**	**AQE**	**Reference**
MOF **1**	LED solar simulator, 100 mW/cm^2	MeOH + AA	-	1635 µmol g^{-1} h^{-1}	-	This work
MOF **2**	LED solar simulator, 100 mW/cm^2	MeOH + AA	-	1437 µmol g^{-1} h^{-1}	2.16 % at 450 nm	This work
Pt/ACM-1	300 W Xe lamp, 130 mW/cm^2	TEA	Pt	67 µmol h^{-1}	0.43 % at 440 nm	[1]
Sc-TBAPy	300 W Xe lamp, $\lambda \geq 420$ nm	TEA	-	32.8 µmol g^{-1} h^{-1}	0.364 % at 450 nm	[2]
Bi-TBAPy	300 W Xe lamp	TEOA	Pt	140 µmol g^{-1} h^{-1}	-	[3]
TBAP-α	300 W Xe lamp $\lambda \geq 420$ nm	AA	Pt	3108 µmol g^{-1} h^{-1}	4.1 % at 420 nm	[4]
Ni-TBAPy-NB	300 W Xe lamp $\lambda \geq 420$ nm	MeOH + AA	-	~5 mmol g^{-1} h^{-1}	8.0 % at 420 nm	[5]
Ni-TBAPy-EF	300 W Xe lamp $\lambda \geq 420$ nm	MeOH + AA	-	17.4 mmol g^{-1} h^{-1}	6.48 % at 460 nm	[6]

TEA = triethylamine; TEOA = triethanolamine; AA = ascorbic acid

References

1. Cadiau, A.; Kolobov, N.; Srinivasan, S.; Goesten, M. G.; Haspel, H.; Bavykina, A. V; Tchalala, M. R.; Maity, P.; Goryachev, A.; Poryvaev, A. S.; Eddaoudi, M.; Fedin, M. V; Mohammed, O. F. and Gascon, J., A Titanium Metal–Organic Framework with Visible-Light-Responsive Photocatalytic Activity, *Angew. Chem. Int. Ed.*, **2020**, *59*, 13468–13472.

2. Kinik, F. P.; Ortega-Guerrero, A.; Ebrahim, F. M.; Ireland, C. P.; Kadioglu, O.; Mace, A.; Asgari, M. and Smit, B., Toward Optimal Photocatalytic Hydrogen Generation from Water Using Pyrene-Based Metal–Organic Frameworks, *ACS Appl. Mater. Interfaces*, **2021**, *13*, 57118–57131.

3. Xiao, Y.; Guo, X.; Liu, J.; Liu, L.; Zhang, F. and Li, C., Development of a bismuth-based metal-organic framework for photocatalytic hydrogen production, *Chinese Journal of Catalysis*, **2019**, *40*, 1339–1344.

4. Aitchison, C. M.; Kane, C. M.; McMahon, D. P.; Spackman, P. R.; Pulido, A.; Wang, X.; Wilbraham, L.; Chen, L.; Clowes, R.; Zwijnenburg, M. A.; Sprick, R. S.; Little, M. A.; Day, G. M. and Cooper, A. I., Photocatalytic proton reduction by a computationally identified, molecular hydrogen-bonded framework, *J. Mater. Chem. A Mater.*, **2020**, *8*, 7158–7170.

5. Liu, L.; Du, S.; Guo, X.; Xiao, Y.; Yin, Z.; Yang, N.; Bao, Y.; Zhu, X.; Jin, S.; Feng, Z. and Zhang, F., Water-Stable Nickel Metal–Organic Framework Nanobelts for Cocatalyst-Free Photocatalytic Water Splitting to Produce Hydrogen, *J. Am. Chem. Soc.*, **2022**, *144*, 2747–2754.

6. Yang, H.; Li, X.; Han, S.; Tian, L.; Liu, J.; Li, Z.; Wang, Y. and Zou, Z., Controllable Regulation of Ligand Stacking in MOF Single-Crystal: From F-F to E-F Stacking Mode for Efficient Photocatalysis, *Adv. Funct. Mater.*, **2025**, *35*, 2501344.
